# Simultaneous Numerical Determination of Two Time-dependent Coefficients in Second Order Parabolic Equation With Nonlocal Initial and Boundary Conditions

**DOI:** 10.12688/f1000research.173252.2

**Published:** 2026-04-03

**Authors:** Mohammed A.J.Al-Shatrah, Mohammed Sabah Hussein

**Affiliations:** 1 Ministry of Education, Directorate of Education Thi Qar, Thi Qar, 64001, Iraq; 2 University of Baghdad Al-Jaderyia Campus College of Science, Baghdad, Baghdad Governorate, Iraq

**Keywords:** Finite difference method, Crank-Nicolson, Tikhonov regularization, Inverse problem, Coefficient problem, Ill-posed problem, Nonlocal conditions, Parabolic equation.

## Abstract

**Background:**

This study establishes a mathematically consistent and computational framework for the simultaneous identification of two time-dependent coefficients in a one-dimensional second-order parabolic partial differential equation. The considered problem is governed by nonlocal initial, boundary, and integral overdetermination conditions.

**Methods:**

The direct problem is solved using the Crank-Nicolson finite difference method (FDM), which ensures unconditional stability and second-order accuracy in both spatial and temporal discretizations. The corresponding inverse problem is reformulated as a nonlinear regularized least-squares optimization problem and efficiently solved using the MATLAB subroutine *lsqnonlin* from the optimization Toolbox. Due to the intrinsic ill-posedness of the inverse formulation, small input data errors lead to big output errors. Then, Tikhonov regularization is employed to enhance numerical stability and robustness.

**Results:**

Extensive numerical experiments are carried out under exact and noisy data to evaluate the numerical accuracy and convergence behavior of the method. The results confirm that the regularization technique effectively damps numerical oscillations, minimizes reconstruction error, and ensures reliable recovery of the unknown coefficients. Sensitivity analysis further reveals the essential role of the regularization parameter in controlling the trade-off between stability and accuracy.

**Conclusions:**

The proposed approach provides an accurate and computationally efficient tool for IP in heat transfer, diffusion processes, and related applied sciences.

## 1. Introduction

Inverse problems (IPs) arise naturally in many scientific and engineering disciplines because these systems are typically modeled by differential equations. In the context of ordinary differential equations, the direct problem refers to obtaining solutions for a given system, whereas IP focuses on reconstructing the governing system from observed characteristics. Historically, the role of IPs was recognized in celestial mechanics. ^1^ In practical applications, IPs are commonly encountered when one seeks to determine unknown causes from their observed outcomes, in contrast to direct problems where effects are predicted from known causes. Compared with direct problems IPs are often more difficult to solve due to their ill-posed nature. The solutions may not exist, may not be unique, or may not depend continuously on the input data. ^2^ Such problems appear in almost every scientific and technological domain, particularly in models derived from social and physical systems. Most of these models are expressed through differential and integral equations. Therefore, their analysis requires not only solving the equations but also interpreting the system behavior under different conditions, provided that sufficient information is available. IPs associated with such equations arise in a wide range of scientific and engineering applications, as well as in the modeling of social processes. Many physical phenomena are described by mathematical models in the form of initial and boundary value problems for partial differential equations (PDEs). Frequently, these models involve differential and integral equations. ^3^ In boundary-type IPs the goal is to recover unknown boundary conditions. This often leads to classical ill-posedness because the absence of continuous dependence of the solution on the input data. Numerical solution (NS) of direct mathematical physics problems is presently a well-studied matter. In solving multi-dimensional boundary value problems, difference methods and the finite element method are widely used. PDEs from the foundation of many applied mathematical models. Their solutions are obtained by considering the govering equations together with additional relations, boundary, and initial conditions, among other elements. ^4^ The following reconstruction problems serve as examples of IP applications in daily life, tomography, initial condition estimation of transient problems in conductive heat transfer, detection of non-metallic materials beneath the surface using reflected radiation methods, intensity, position estimation of illuminated radiation from a biological source using experimental radiation measures, and thermal source intensity estimation with functional dependence in space. ^5– 9^ On the other hand, there are three different kinds of differential equations, Second-order equations are the most crucial for applications, Elliptic, Parabolic, and hyperbolic equations are examples of these equations. Yuldashev and other authors studied elliptic type integrate differential equations in ^10, 11^ while hyperbolic and parabolic were studied in ^12– 14^ and ^15– 17^ respectively. The alternating direction explicit method used for reconstructing solutions is also efficient and unconditionally stable, in ^18^ is restructured into a nonlinear regularized least-square optimization problem, and is effectively resolved using the MATLAB subroutine *lsqnonlin* from the optimization toolbox. The Crank-Nicolson (C-N) FDM together with the TR was used in ^19– 21^ the effectiveness of the computational method was shown together with the proof of the solution’s existence and uniqueness. To find a stable and accurate approximate solution of finite differences. Studies in ^22– 26^ show that IPs with a time-dependent source coefficient in heat equations acknowledge a smooth solution pair when data are available at an observation point. Moreover, providing an explicit formula for the time-dependent coefficient improves the understanding of the problem behavior. Recently, several studies have addressed inverse coefficient problems for parabolic equations with nonlocal conditions and overdetermination data. For instance, Huzyk et al. (2023) ^27^ investigated coefficient identification for strongly degenerate parabolic equations, while Azizbayov and Safarova (2025) ^28^ studied IPs for parabolic equations with nonlocal boundary conditions and two-point overdetermination. These studies further demonstrate the importance of developing stable numerical techniques for such IPs. This paper’s IP classical solution for a second order parabolic equation has been shown to be exist and unique by Elvin Azizbayov. ^29^ As a result, the main objective of the current work is a numerical realization of such a problem. This paper is organized into five sections Section 2, includes a mathematical formulation of the IP under study. Section 3, a numerical method for solving the forward problem using C-N FDM. In Section 4 the IPs mathematical solution is shown with an initial guess, while in Section 5, discuss the numerical problem and explain the obtained results.

## 2. Mathematical formulation

As a mathematical model, we consider $D_{T}$ be a rectangular region that is defined by $D_{T}:\left\{\;\left(v,\tau \right):0\;\leq \;v\;\leq \;1,\;0\;\leq \;\tau \;\leq \;T\;\right\}\;$ and (T>0), be a fixed number, the one-dimensional IP of determining of unknown functions {w(v,τ),a(τ),b(τ)} for the upcoming parabolic equation,

$$f\left(v,\tau \right)=c\left(\tau \right)w_{\tau }\left(v,\tau \right)-w_{\mathrm{vv}}\left(v,\tau \right)-a\left(\tau \right)w\left(v,\tau \right)-b\left(\tau \right)g\left(v,\tau \right),\;\left(v,\tau \right)\;\in \;D_{T},$$ (1)

with, the nonlocal initial conditions (ICs)

$$w\left(v,0\right)+\mathrm{\delta w}\left(v,T\right)+\int_{0}^{T}\hat{Ƥ}\left(\tau \right)w\left(v,\tau \right)\mathrm{d\tau}=\zeta \left(v\right),\;v\;\in \;\left[0,1\right],$$ (2)

The problem is considered with periodic boundary condition in the spatial variable *v* on the interval [0,1].

w(0,τ)=γw(1,τ),τ∈[0,T], (3)

and, nonlocal integral condition

$$\int_{0}^{1}w\left(v,\tau \right)\mathrm{dv}=0,\;\tau \;\in \;\left[0,T\right],$$ (4)

and the overdetermination conditions

$$w\left(v_{i},\tau \right)=z_{i}\left(\tau \right)\;\left(i=1,2\right),\tau \;\in \;\left[0,T\right],$$ (5)

where, $\gamma ,\delta \;\geq \;0,v_{i}\;\in \;\left(0,1\right),\left(i=1,2\right);v_{1}\neq v_{2}\;$ , are fixed numbers, $c\left(\tau \right)>0,g\left(v,\tau \right),f\left(v,\tau \right),\hat{Ƥ}\left(\tau \right)\;\geq \;0,\zeta \left(v\right),z_{i}\left(\tau \right)\;\left(i=1,2\right)$ are given functions, w(v,τ),a(τ),b(τ) are the sought functions ( v is space component and τ is time component).

### 2.1 Definition 1

Consider the triplet {w(v,τ),a(τ),b(τ)} represents a classical solution, to the IP (1)−(5), if the functions w(v,τ),a(τ), and b(τ) satisfies the following conditions:

1) The function w(v,τ) and its derivatives $w_{\tau }\left(v,\tau \right),w_{v}\left(v,\tau \right),w_{\mathrm{vv}}\left(v,\tau \right)$ are continuous in the domain $D_{T}.$

2) The functions a(τ), and b(τ) are continuous on [0,T].

3) Equation (1) and conditions (2)−(5) are satisfied in the classical (usual) sense.

### 2.2 Existence of the inverse problem classical solution

**2.2.1 Theorem 1[27]:** Let the assumptions (μ1)−(μ4) and the condition

((E(T)+2)H(T)+G(T)+Q(T))(E(T)+2)<1,

where,

$$\mu 1)\;\zeta \left(v\right)\;\in \;C^{2}\left[0,1\right],\zeta^{\left(3\right)}\left(v\right)\;\in \;L_{2}\left(0,1\right),\zeta \left(0\right)=\gamma \zeta \left(1\right),\zeta^{\prime }\left(0\right)=\zeta^{\prime }\left(1\right),\zeta^{\prime \prime }\left(0\right)={\gamma \zeta }^{\prime \prime }\left(1\right);$$

where, $\hat{a}=\frac{1-\gamma }{1+\gamma }$ and $\hat{b}=\frac{\gamma }{1+\gamma }$ ; γ≠±1.

$$\mu 2)\;f\left(v,\tau \right)\;\in \;C_{v,\tau }^{2,0}\left(D_{T}\right),f_{\mathrm{vvv}}\left(v,\tau \right)\;\in \;L_{2}\left(D_{T}\right),f\left(0,\tau \right)=\mathrm{\gamma f}\left(1,\tau \right),f_{v}\left(0,\tau \right)=f_{v}\left(1,\tau \right),f_{\mathrm{vv}}\left(0,\tau \right)=\gamma f_{\mathrm{vv}}\left(1,\tau \right),\left(\gamma \neq \pm 1\right),0\;\leq \;\tau \;\leq \;T.$$

$$\mu 3)\;g\left(v,\tau \right)\;\in \;C_{v,\tau }^{2,0}\left(D_{T}\right),g_{\mathrm{vvv}}\left(v,\tau \right)\;\in \;L_{2}\left(D_{T}\right),g\left(0,\tau \right)=\mathrm{\gamma g}\left(1,\tau \right),g_{v}\left(0,\tau \right)=g_{v}\left(1,\tau \right),g_{\mathrm{vv}}\left(0,\tau \right)=\gamma g_{\mathrm{vv}}\left(1,\tau \right),\left(\gamma \neq \pm 1\right),0\;\leq \;\tau \;\leq \;T.$$

$$\mu 4)\;\delta \;\geq \;0,c\left(\tau \right)\;\in \;C\left[0,T\right],c\left(\tau \right)>0,0\;\leq \;\hat{Ƥ}\left(\tau \right)\;\in \;C\left[0,T\right],z_{i}\left(\tau \right)\;\in \;C^{1}\left[0,T\right],$$

$$z\left(\tau \right)=z_{1}\left(\tau \right)g\left(v_{2},\tau \right)-z_{2}\left(\tau \right)g\left(v_{1},\tau \right)\neq 0,\left(i=1,2\right),0\;\leq \;\tau \;\leq \;T.$$

where,

$$\;E_{1}\left(T\right)=2{\left(1+\delta \right)}^{-1}{\left\|\zeta \left(v\right)\right\|}_{L_{2}\left(0,1\right)}+2\sqrt{T}\left(1+\delta {\left(1+\delta \right)}^{-1}\right){\left\|\frac{1}{c\left(\tau \right)}\right\|}_{C\left[0,T\right]}{\left\|f\left(v,\tau \right)\right\|}_{L_{2}\left(D_{T}\right)}),$$ (6)

$$E_{2}\left(T\right)=2\sqrt{10}{\left\|\zeta^{\left(3\right)}\left(v\right)\right\|}_{L_{2}\left(0,1\right)}+2\sqrt{10T}\left(1+\delta \right){\left\|\frac{1}{c\left(\tau \right)}\right\|}_{C\left[0,T\right]}{\left\|f_{\mathrm{vvv}}\left(v,\tau \right)\right\|}_{L_{2}\left(D_{T}\right)},$$ (7)

$$\begin{array}{c} E_{3}\left(T\right)=4\sqrt{3}{\left\|\zeta^{\left(3\right)}\left(v\right)\left(1-\hat{b}-\hat{a}v\right)-3\hat{a}\zeta^{\left(2\right)}\left(v\right)\right\|}_{L_{2}\left(0,1\right)}+4\sqrt{3T}\left(1+\delta \right){\left\|\frac{1}{c\left(\tau \right)}\right\|}_{C\left[0,T\right]}\\ {\left\|f_{\mathrm{vvv}}\left(v,\tau \right)\left(1-\hat{b}-\hat{a}v\right)-3\hat{a}f_{\mathrm{vv}}\left(v,\tau \right)\right\|}_{L_{2}\left(D_{T}\right)}+12\sqrt{2}\left|\hat{a}\right|\left(1+\delta \right){\left\|\frac{1}{c\left(\tau \right)}\right\|}_{C\left[0,T\right]}{\left\|\zeta^{\left(3\right)}\left(v\right)\right\|}_{L_{2}\left(0,1\right)}\\ +12\sqrt{3}\left|\hat{a}\right|{\left\|\frac{1}{c\left(\tau \right)}\right\|}_{C\left[0,T\right]}^{2}\sqrt{{\left\|c\left(\tau \right)\right\|}_{C\left[0,T\right]}}T\sqrt{T}{\left(1+\delta \right)}^{2}{\left\|f_{\mathrm{vvv}}\left(v,\tau \right)\right\|}_{L_{2}\left(D_{T}\right)}, \end{array}$$ (8)

$$H_{1}\left(T\right)=\left(1+\delta {\left(1+\delta \right)}^{-1}\right)T{\left\|\frac{1}{c\left(\tau \right)}\right\|}_{C\left[0,T\right]},$$ (9)

$$H_{2}\left(T\right)=\sqrt{6}T\left(1+\delta \right){\left\|\frac{1}{c\left(\tau \right)}\right\|}_{C\left[0,T\right]},$$ (10)

$$H_{3}\left(T\right)=\sqrt{6}T\left(1+\delta \right){\left\|\frac{1}{c\left(\tau \right)}\right\|}_{C\left[0,T\right]}+6\sqrt{2}\left|\hat{a}\right|{\left\|\frac{1}{c\left(\tau \right)}\right\|}_{C\left[0,T\right]}^{2}\sqrt{{\left\|c\left(\tau \right)\right\|}_{C\left[0,T\right]}}T^{2}{\left(1+\delta \right)}^{2},$$ (11)

$$G_{1}\left(T\right)={\left(1+\delta \right)}^{-1}T{\left\|\hat{Ƥ}\left(\tau \right)\right\|}_{C\left[0,T\right]},$$ (12)

$$G_{2}\left(T\right)=\sqrt{6}T{\left\|\hat{Ƥ}\left(\tau \right)\right\|}_{C\left[0,T\right]},$$ (13)

$$G_{3}\left(T\right)=\sqrt{3}T{\left\|\hat{Ƥ}\left(\tau \right)\right\|}_{C\left[0,T\right]}\left[\sqrt{2}+4\left|\hat{a}\right|{\left\|\frac{1}{c\left(\tau \right)}\right\|}_{C\left[0,T\right]}\left(1+\delta \right)\right],$$ (14)

$$Q_{1}\left(T\right)=\left(1+\delta {\left(1+\delta \right)}^{-1}\right){\left\|\frac{1}{c\left(\tau \right)}\right\|}_{C\left[0,T\right]}\sqrt{T}{\left\|b\left(\tau \right)\right\|}_{C\left[0,T\right]}{\left\|g\left(v,\tau \right)\right\|}_{L_{2}\left(D_{T}\right)},$$ (15)

$$Q_{2}\left(T\right)=4\sqrt{3T}\left(1+\delta \right){\left\|\frac{1}{c\left(\tau \right)}\right\|}_{C\left[0,T\right]}{\left\|g_{\mathrm{vvv}}\left(v,\tau \right)\right\|}_{L_{2}\left(D_{T}\right)},$$ (16)

Q3(T)=62T(1+δ)‖1c(τ)‖C[0,T](‖gvvv(v,τ)(1−b^−a^v)−3a^gvv(v,τ)‖L2(DT)+|a^|‖1c(τ)‖C[0,T]‖c(τ)‖C[0,T]T(1+δ)‖gvvv(v,τ)‖L2(DT)), (17)

where,

$$\;E_{4}\left(T\right)=E_{1}\left(T\right)+E_{2}\left(T\right)+E_{3}\left(T\right),H_{4}\left(T\right)=H_{1}\left(T\right)+H_{2}\left(T\right)+H_{3}\left(T\right),$$

$$G_{4}\left(T\right)=G_{1}\left(T\right)+G_{2}\left(T\right)+G_{3}\left(T\right),\;\text{and},\;Q_{4}\left(T\right)=Q_{1}\left(T\right)+Q_{2}\left(T\right)+Q_{3}\left(T\right),$$

$$\;E_{5}\left(T\right)={\left\|{\left[z\left(\tau \right)\right]}^{-1}\right\|}_{C\left[0,T\right]}\left\{{\left\|z_{1}\left(\tau \right)\left(c\left(\tau \right){z_{2}}^{\prime }\left(\tau \right)-f\left(v_{2},\tau \right)\right)-z_{2}\left(\tau \right)\left(c\left(\tau \right){z_{1}}^{\prime }\left(\tau \right)-f\left(v_{1},\tau \right)\right)\right\|}_{C\left[0,T\right]}+{\left(\sum_{k=1}^{\infty }{\lambda_{k}}^{-2}\right)}^{\frac{1}{2}}\left[2\sqrt{2}\;[\left(1+4\sqrt{3}\left|\hat{a}\right|{\left\|\frac{1}{c\left(\tau \right)}\right\|}_{C\left[0,T\right]}\left(1+\delta \right)\right){\left\|\zeta^{\left(3\right)}\left(v\right)\right\|}_{L_{2}\left(0,1\right)}+{\left\|\zeta^{\left(3\right)}\left(v\right)\left(1-\hat{b}-\hat{a}v\right)-3\hat{a}\zeta^{\left(2\right)}\left(v\right)\right\|}_{L_{2}\left(0,1\right)}\right]+2\sqrt{2T}\left(1+\delta \right){\left\|\frac{1}{c\left(\tau \right)}\right\|}_{C\left[0,T\right]}\left[\left(1+6\sqrt{2}\left|\hat{a}\right|{\left\|\frac{1}{c\left(\tau \right)}\right\|}_{C\left[0,T\right]}\sqrt{{\left\|c\left(\tau \right)\right\|}_{C\left[0,T\right]}}T\left(1+\delta \right)\right){\left\|f_{\mathrm{vvv}}\left(v,\tau \right)\right\|}_{L_{2}\left(D_{T}\right)})+{\left\|f_{\mathrm{vvv}}\left(v,\tau \right)\left(1-\hat{b}-\hat{a}v\right)-3\hat{a}f_{\mathrm{vv}}\left(v,\tau \right)\right\|}_{L_{2}\left(D_{T}\right)}]\right]{\left\|\left|g\left(v_{2},\tau \right)\right|+\left|g\left(v_{1},\tau \right)\right|\right\|}_{C\left[0,T\right]}{\left\|\hat{a}v+\hat{b}\right\|}_{C\left[0,1\right]}\right\},$$ (18)

$$E_{6}\left(T\right)={\left\|{\left[z\left(\tau \right)\right]}^{-1}\right\|}_{C\left[0,T\right]}\left\{{\left\|g\left(v_{2},\tau \right)\left(c\left(\tau \right){z_{1}}^{\prime }\left(\tau \right)-f\left(v_{1},\tau \right)\right)-g\left(v_{1},\tau \right)\left(c\left(\tau \right){z_{2}}^{\prime }\left(\tau \right)-f\left(v_{2},\tau \right)\right)\right\|}_{C\left[0,T\right]}+{\left(\sum_{k=1}^{\infty }{\lambda_{k}}^{-2}\right)}^{\frac{1}{2}}\left[2\sqrt{2}\;[\left(1+4\sqrt{3}\left|\hat{a}\right|{\left\|\frac{1}{c\left(\tau \right)}\right\|}_{C\left[0,T\right]}\left(1+\delta \right)\right){\left\|\zeta^{\left(3\right)}\left(v\right)\right\|}_{L_{2}\left(0,1\right)}+{\left\|\zeta^{\left(3\right)}\left(v\right)\left(1-\hat{b}-\hat{a}v\right)-3\hat{a}\zeta^{\left(2\right)}\left(v\right)\right\|}_{L_{2}\left(0,1\right)}\right]+2\sqrt{2T}\left(1+\delta \right){\left\|\frac{1}{c\left(\tau \right)}\right\|}_{C\left[0,T\right]}\left[\left(1+6\sqrt{2}\left|\hat{a}\right|{\left\|\frac{1}{c\left(\tau \right)}\right\|}_{C\left[0,T\right]}\sqrt{{\left\|c\left(\tau \right)\right\|}_{C\left[0,T\right]}}T\left(1+\delta \right)\right){\left\|f_{\mathrm{vvv}}\left(v,\tau \right)\right\|}_{L_{2}\left(D_{T}\right)})+{\left\|f_{\mathrm{vvv}}\left(v,\tau \right)\left(1-\hat{b}-\hat{a}v\right)-3\hat{a}f_{\mathrm{vv}}\left(v,\tau \right)\right\|}_{L_{2}\left(D_{T}\right)}]\right]х{\left\|\left|z_{1}\left(\tau \right)\right|+\left|z_{2}\left(\tau \right)\right|\right\|}_{C\left[0,T\right]}{\left\|\hat{a}v+\hat{b}\right\|}_{C\left[0,1\right]}\right\},$$ (19)

$$H_{5}\left(T\right)=2T{\left\|{\left[z\left(\tau \right)\right]}^{-1}\right\|}_{C\left[0,T\right]}{\left(\sum_{k=1}^{\infty }{\lambda_{k}}^{-2}\right)}^{\frac{1}{2}}\left(1+\delta \right){\left\|\frac{1}{c\left(\tau \right)}\right\|}_{C\left[0,T\right]}\left(1+3\sqrt{2}\left|\hat{a}\right|{\left\|\frac{1}{c\left(\tau \right)}\right\|}_{C\left[0,T\right]}\sqrt{{\left\|c\left(\tau \right)\right\|}_{C\left[0,T\right]}}T\left(1+\delta \right)\right){\left\|\left|g\left(v_{2},\tau \right)\right|+\left|g\left(v_{1},\tau \right)\right|\right\|}_{C\left[0,T\right]}{\left\|\hat{a}v+\hat{b}\right\|}_{C\left[0,1\right]},$$ (20)

$$H_{6}\left(T\right)=2T{\left\|{\left[z\left(\tau \right)\right]}^{-1}\right\|}_{C\left[0,T\right]}{\left(\sum_{k=1}^{\infty }{\lambda_{k}}^{-2}\right)}^{\frac{1}{2}}\left(1+\delta \right){\left\|\frac{1}{c\left(\tau \right)}\right\|}_{C\left[0,T\right]}\left(1+3\sqrt{2}\left|\hat{a}\right|{\left\|\frac{1}{c\left(\tau \right)}\right\|}_{C\left[0,T\right]}\sqrt{{\left\|c\left(\tau \right)\right\|}_{C\left[0,T\right]}}T\left(1+\delta \right)\right){\left\|\left|z_{1}\left(\tau \right)\right|+\left|z_{2}\left(\tau \right)\right|\right\|}_{C\left[0,T\right]}{\left\|\hat{a}v+\hat{b}\right\|}_{C\left[0,1\right]},$$ (21)

$$G_{5}\left(T\right)=2T{\left\|{\left[z\left(\tau \right)\right]}^{-1}\right\|}_{C\left[0,T\right]}{\left(\sum_{k=1}^{\infty }{\lambda_{k}}^{-2}\right)}^{\frac{1}{2}}{\left\|\hat{Ƥ}\left(\tau \right)\right\|}_{C\left[0,T\right]}\left(1+2\sqrt{3}(1+\delta \right){\left\|\frac{1}{c\left(\tau \right)}\right\|}_{C\left[0,T\right]}){\left\|\left|g\left(v_{2},\tau \right)\right|+\left|g\left(v_{1},\tau \right)\right|\right\|}_{C\left[0,T\right]}{\left\|\hat{a}v+\hat{b}\right\|}_{C\left[0,1\right]},$$ (22)

$$G_{6}\left(T\right)=2T{\left\|{\left[z\left(\tau \right)\right]}^{-1}\right\|}_{C\left[0,T\right]}{\left(\sum_{k=1}^{\infty }{\lambda_{k}}^{-2}\right)}^{\frac{1}{2}}{\left\|\hat{Ƥ}\left(\tau \right)\right\|}_{C\left[0,T\right]}\left(1+2\sqrt{3}(1+\delta \right){\left\|\frac{1}{c\left(\tau \right)}\right\|}_{C\left[0,T\right]}){\left\|\left|z_{1}\left(\tau \right)\right|+\left|z_{2}\left(\tau \right)\right|\right\|}_{C\left[0,T\right]}{\left\|\hat{a}v+\hat{b}\right\|}_{C\left[0,1\right]},$$ (23)

$$Q_{5}\left(T\right)=2\sqrt{2T}\left(1+\delta \right){\left\|\frac{1}{c\left(\tau \right)}\right\|}_{C\left[0,T\right]}{\left\|{\left[z\left(\tau \right)\right]}^{-1}\right\|}_{C\left[0,T\right]}{\left(\sum_{k=1}^{\infty }{\lambda_{k}}^{-2}\right)}^{\frac{1}{2}}[\left(1+6\sqrt{2}\left|\hat{a}\right|{\left\|\frac{1}{c\left(\tau \right)}\right\|}_{C\left[0,T\right]}\sqrt{{\left\|c\left(\tau \right)\right\|}_{C\left[0,T\right]}}T\left(1+\delta \right)\right){\left\|g_{\mathrm{vvv}}\left(v,\tau \right)\right\|}_{L_{2}\left(D_{T}\right)}+{\left\|g_{\mathrm{vvv}}\left(v,\tau \right)\left(1-\hat{b}-\hat{a}x\right)-3\hat{a}g_{\mathrm{vv}}\left(v,\tau \right)\right\|}_{L_{2}\left(D_{T}\right)}{\left\|\left|g\left(v_{2},\tau \right)\right|+\left|g\left(v_{1},\tau \right)\right|\right\|}_{C\left[0,T\right]}{\left\|\hat{a}v+\hat{b}\right\|}_{C\left[0,1\right]},$$ (24)

$$Q_{6}\left(T\right)=2\sqrt{2T}\left(1+\delta \right){\left\|\frac{1}{c\left(\tau \right)}\right\|}_{C\left[0,T\right]}{\left\|{\left[z\left(\tau \right)\right]}^{-1}\right\|}_{C\left[0,T\right]}{\left(\sum_{k=1}^{\infty }{\lambda_{k}}^{-2}\right)}^{\frac{1}{2}}[\left(1+6\sqrt{2}\left|\hat{a}\right|{\left\|\frac{1}{c\left(\tau \right)}\right\|}_{C\left[0,T\right]}\sqrt{{\left\|c\left(\tau \right)\right\|}_{C\left[0,T\right]}}T\left(1+\delta \right)\right){\left\|g_{\mathrm{vvv}}\left(v,\tau \right)\right\|}_{L_{2}\left(D_{T}\right)}+{\left\|g_{\mathrm{vvv}}\left(v,\tau \right)\left(1-\hat{b}-\hat{a}x\right)-3\hat{a}g_{\mathrm{vv}}\left(v,\tau \right)\right\|}_{L_{2}\left(D_{T}\right)}{\left\|\left|z_{1}\left(\tau \right)\right|+\left|z_{2}\left(\tau \right)\right|\right\|}_{C\left[0,T\right]}{\left\|\hat{a}v+\hat{b}\right\|}_{C\left[0,1\right]},$$ (25)

where,

$$E\left(T\right)=E_{4}\left(T\right)+E_{5}\left(T\right)+E_{6}\left(T\right),H\left(T\right)=H_{4}\left(T\right)+H_{5}\left(T\right)+H_{6}\left(T\right),G\left(T\right)=G_{4}\left(T\right)+G_{5}\left(T\right)+G_{6}\left(T\right),\text{and}\;Q\left(T\right)=Q_{4}\left(T\right)+Q_{5}\left(T\right)+Q_{6}\left(T\right),$$

holds, then problem (1)−(5) , has a unique solution in the ball $K=K_{R},$ of the space ${X_{k}}^{3}$ , where, $\lambda_{k}=2\mathrm{k\pi};\left(k=0,1,\ldots \right).$

## 3. FDM scheme for direct (Forward) problem

The direct problem is solved in this section, where the coefficients a(τ) , c(τ) and b(τ) are assumed to be given. To solve this problem, the Crank-Nicolson (C-N) FDM scheme is used to compute the solution of the nonlocal problem given by Equations (1)– (4) . Where the domain $D_{T}$ had been divided into M×N mesh with spatial step size $∆v=\frac{1}{M}$ and the time step size $∆\tau =\frac{T}{N}$ where M and N are given positive integers. The grid points have been given by

$$v_{i}=i∆v,\;i=\bar{0,M,}$$ (26)

$$\tau_{j}=j∆\tau ,\;j=\bar{0,N,}$$ (27)

These quantities’ discretized form is given as follows,

$$w\left(v_{i},\tau_{j}\right)\;≕\;w_{i,j},a\left(\tau_{j}\right)\;≕\;a_{j},b\left(\tau_{j}\right)\;≕\;b_{j},c\left(\tau_{j}\right)\;≕\;c_{j},f\left(v_{i},\tau_{j}\right)\;≕\;f_{i,j},g\left(v_{i},\tau_{j}\right)\;≕\;g_{i,j},\text{and}\;\zeta \left(v_{i}\right)\;≕\;\zeta_{i}\;\text{for}\;i=\bar{0,M,}\;j=\bar{0,N.}\;$$

Applying the C-N FDM, for the discretizes Equation (1) , which approximated as,

$$\frac{w_{i,j+1}-w_{i,j}}{∆\tau }=\frac{1}{2}\left({\tilde{d}}_{j}\frac{w_{i+1,j}-2w_{i,j}+w_{i-1,j}}{{\left(∆v\right)}^{2}}+{\tilde{a}}_{j}w_{i,j}+{\tilde{b}}_{j}g_{i,j}+{\tilde{d}}_{j}f_{i,j}+{\tilde{d}}_{j+1}\frac{w_{i+1,j+1}-2w_{i,j+1}+w_{i-1,j+1}}{{\left(∆v\right)}^{2}}+{\tilde{a}}_{j+1}w_{i,j+1}+{\tilde{b}}_{j+1}g_{i,j+1}+{\tilde{d}}_{j+1}f_{i,j+1}\right),\;i=\bar{1,M,}\;j=\bar{0,N,}$$ (28)

where, ${\tilde{d}}_{j}=\frac{1}{c_{j}}$ , ${\tilde{a}}_{j}=\frac{a_{j}}{c_{j}}$ , ${\tilde{b}}_{j}=\frac{b_{j}}{c_{j}}$ , ${\tilde{d}}_{j+1}=\frac{1}{c_{j+1}}$ , ${\tilde{a}}_{j+1}=\frac{a_{j+1}}{c_{j+1}}$ , and, ${\tilde{b}}_{j+1}=\frac{b_{j+1}}{c_{j+1}}$ simplifying the above Equation (28), get the following difference equation,

$$-{\hat{A}}_{j+1}w_{i-1,j+1}+\left(1+{\hat{E}}_{j+1}\right)w_{i,j+1}-{\hat{A}}_{j+1}w_{i+1,j+1}={\hat{A}}_{j}w_{i-1,j}+\left(1-{\hat{E}}_{j}\right)w_{i,j}+{\hat{A}}_{j}w_{i+1,j}+\frac{∆\tau }{2}\hat{H}+\frac{∆\tau }{2}\hat{D},$$ (29)

where, ${\hat{A}}_{j}=\frac{∆\tau {\tilde{d}}_{j}}{2{\left(∆v\right)}^{2}}$ , ${\hat{A}}_{j+1}=\frac{∆\tau {\tilde{d}}_{j+1}}{2{\left(∆v\right)}^{2}}$ , ${\hat{E}}_{j+1}=\left(2{\hat{A}}_{j+1}-\frac{∆\tau }{2}{\tilde{a}}_{j+1}\right)$ , ${\hat{E}}_{j}=\left(2{\hat{A}}_{j}-\frac{∆\tau }{2}{\tilde{a}}_{j}\right)$ , $\hat{H}={\tilde{b}}_{j}g_{i,j}+{\tilde{b}}_{j+1}g_{i,j+1}$ , and, $\hat{D}={\tilde{d}}_{j}f_{i,j}+{\tilde{d}}_{j+1}f_{i,j+1}$ .

The discretization form of (29) is

$$F\left(\underline{a}\right)=\sum_{i=1}^{2}\sum_{j=1}^{N}{\left[w\left(v_{i},\tau_{j}\right)-z_{i}\left(\tau_{j}\right)\right]}^{2}+\beta \left(\sum_{j=1}^{N}{a_{j}}^{2}+\sum_{j=1}^{N}{b_{j}}^{2}\right).$$ (30)

The C-N FDM discretizes Equations (2)–(4) which approximated as,

$$w_{i,0}+\delta w_{i,j}+I\left(v_{i}\right)=\zeta_{i},\;i=\bar{1,M,}$$ (31)

where $I\left(v_{i}\right)$ is integral in Equation (2), and we have,

$$I\left(v_{i}\right)=\frac{∆\tau }{2}\left(\hat{Ƥ}\left(\tau_{0}\right)w\left(v_{i},\tau_{0}\right)+\hat{Ƥ}\left(\tau_{N}\right)w\left(v_{i},\tau_{N}\right)+2\sum_{j=1}^{N-1}\hat{Ƥ}\left(\tau_{j}\right)w\left(v_{i},\tau_{j}\right)\right),$$

Finally, the trapezoidal rule and C-N discretizes integral condition ( 4 ) as;

$$\;w\left(v_{0},\tau_{j}\right)+w\left(v_{M},\tau_{j}\right)+2\sum_{i=1}^{M-1}w\left(v_{i},\tau_{j}\right)=0,\;i=\bar{0,M}$$ (32)

$$w_{i,0}=\zeta_{i},\;i=\bar{1,M,}\;j=0,$$ (33)

$$\;w_{0,j}=\gamma w_{1,j},\;j=\bar{0,N-1,}\;i=0,$$ (34)

$$L={\left[\begin{array}{cccccccccc} 1 & 0 & 0 & 0 & 0 & \ldots & 0 & 0 & 0 & -2\\ -{\hat{A}}_{j+1} & 1+{\hat{E}}_{j+1} & -{\hat{A}}_{j+1} & 0 & 0 & \ldots & 0 & 0 & 0 & 0\\ 0 & -{\hat{A}}_{j+1} & 1+{\hat{E}}_{j+1} & -{\hat{A}}_{j+1} & 0 & \ldots & 0 & 0 & 0 & 0\\ \ldots & \ldots & \ldots & \ddots & \ddots & \ddots & \vdots & \ldots & \vdots & \vdots \\ 0 & 0 & 0 & 0 & 0 & \ldots & 0 & -{\hat{A}}_{j+1} & 1+{\hat{E}}_{j+1} & -{\hat{A}}_{j+1}\\ 1 & 2 & 2 & 2 & 2 & \ldots & 2 & 2 & 2 & 1 \end{array}\right]}_{\left(M\right)х\left(M\right)},$$

with,

L1=[0A^jw1,j+(1−E^j)w2,j+A^jw3,jA^jw2,j+(1−E^j)w3,j+A^jw4,jA^jw3,j+(1−E^j)w4,j+A^jw5,j⋮A^jwM−2,j+(1−E^j)wM−1,j+A^jwM,jA^jwM−1,j+(1−E^j)wM,j+A^jwM+1,j0],

and, $\hat{V}=\left(\hat{H}+\hat{D}\right)$ , where,

V^=[0(bjg2,j+bj+1g2,j+1)+(d~jf2,j+d~j+1f2,j+1)(bjg3,j+bj+1g3,j+1)+(d~jf3,j+d~j+1f3,j+1)(bjg4,j+bj+1g4,j+1)+(d~jf4,j+d~j+1f4,j+1)⋮(bjgM−1,j+bj+1gM−1,j+1)+(d~jfM−1,j+d~j+1fM−1,j+1)(bjgM,j+bj+1gM,j+1)+(d~jfM,j+d~j+1fM,j+1)0]

$$W_{j+1}={\left[\;w_{1,j+1},w_{2,j+1},\ldots ,w_{M+1,j+1},w_{M,j+1}\;\right]}^{T},W_{j}={\left[\;w_{1,j},w_{2,j},\ldots ,w_{M-1,j},w_{M,j}\;\right]}^{T},\;j=\bar{0,N-1,}$$

From Equation (29) , with the nonlocal initial conditions $w\left(v,0\right)=\zeta \left(v_{i}\right),$ (assuming Ƥ(τ)=δ=0 ), and using Equations (31)– (34), with each time step $\tau_{j}$ , we can be described in matrix form as

$$LW_{j+1}=L1W_{j}+\frac{∆\tau }{2}\hat{V},i=\bar{1,M},j=\bar{0,N-1,}$$ (35)

the Equation (4) desired output data will be approximated using trapezoidal rule as

$$0=\int_{0}^{1}H\left(v\right)w\left(v,\tau_{j}\right)\mathrm{dv}=\frac{∆\tau }{2}\left(w\left(v_{0},\tau_{j}\right)+w\left(v_{M},\tau_{j}\right)+2\sum_{k=1}^{M-1}w\left(v_{k},\tau_{j}\right)\right).$$ (36)

### 3.1 Stability and convergence analysis

The adopted C-N finite difference scheme used to solve the direct problem is unconditionally stable and second order accurate in both spatial and temporal discretization. A brief von Neumann stability analysis shows that the amplification factor satisfies |G|=1 , for all mesh ratios, which guarantees the unconditional stability of the scheme. The local truncation error is of order $\vartheta \left({\left(∆v\right)}^{2},{\left(∆\tau \right)}^{2}\right)$ confirming that the method provides second order accuracy and reliable convergence toward the analytical solution as the mesh is refined. Hence, the C-N formulation ensures both numerical stability and accuracy for the present problem.

The Crank-Nicolson finite difference scheme is well known for its unconditional stability and second-order accuracy when applied to parabolic PDEs. The stability and convergence properties of this scheme have been rigorously studied in the literature (see, for example, Samarskii and Vabishchevich ^4^ and Lesnic ^2^). Therefore the proposed numerical formulation inherits these well-established theoretical properties, ensuring reliable and stable numerical reconstruction for the present IP.

### 3.2 Examples for direct problem

**3.2.1 Example 1:** Assume an illustration for the direct problem given by (1)−(4) with $D_{T}:\left\{\;0\;\leq \;v\;\leq \;1,0\;\leq \;\tau \;\leq \;1\;\right\},$ and the input data are,

$$f\left(v,\tau \right)=\frac{-1}{6}e^{\tau }\left(1+\tau \right)v^{3}{\left(2v-1\right)}^{3}\left(3v-1\right)+\frac{1}{44}\left(133+1188e^{3\tau }+176\pi^{2}\left(3\tau -1\right)-\tau \left(6\tau +1\right)\right)\mathrm{Sin}\left(2\mathrm{\pi v}\right),z\left(\tau \right)=\frac{17}{256}e^{\tau }\left(3\tau -1\right),c\left(\tau \right)=1+9e^{3\tau },\;\tau \;\in \;\left[0,1\right],\;v\;\in \;\left[0,1\right],$$

and, the analytical solution is,

$$w\left(v,\tau \right)=\left(3\tau -1\right)\mathrm{Sin}\left(2\mathrm{\pi v}\right),g\left(v,\tau \right)=e^{\tau }{\left(2v^{2}-v\right)}^{3}\left(3v-1\right),a\left(\tau \right)=\frac{1}{44}\left(1+2\tau \right),\;\text{with},\;b\left(\tau \right)=\frac{1}{6}\left(1+\tau \right),\left(v_{1},v_{2}\right)=\left(\frac{1}{4},\frac{3}{4}\right),\;\tau \;\in \;\left[0,T\right],\left(v,\tau \right)\;\in \;D_{T}\;$$

**Figure 1.**
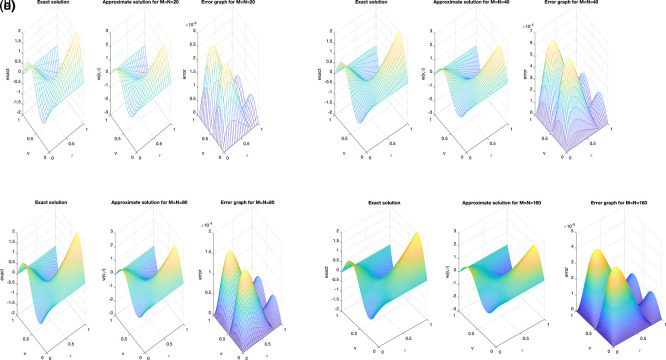
The graphs showing analytical and computational distribution with absolute error for the direct problem (1)−(4) , for example1 (a1)−(d1), represent the size of mesh N=M={20,40,80,160} , respectively.

We investigate the accuracy of the solution using diverse mesh grid size, M=N∈{20,40,80,160} . In Figure 1 (a)–(d), an excellent agreement between the exact and numerical solutions can be clearly observed, indicating high accuracy. It can be noted that as the number of mesh points increases, the accuracy of the obtained solution improves, which reflects the convergence and stability of the proposed numerical scheme. Moreover, the absolute error graph at each mesh shows that the maximum magnitude of error didn’t exceed $\left(10^{-5}\right)$ , as depicted in Figure 1 (a)–(d).

**Figure 2.**
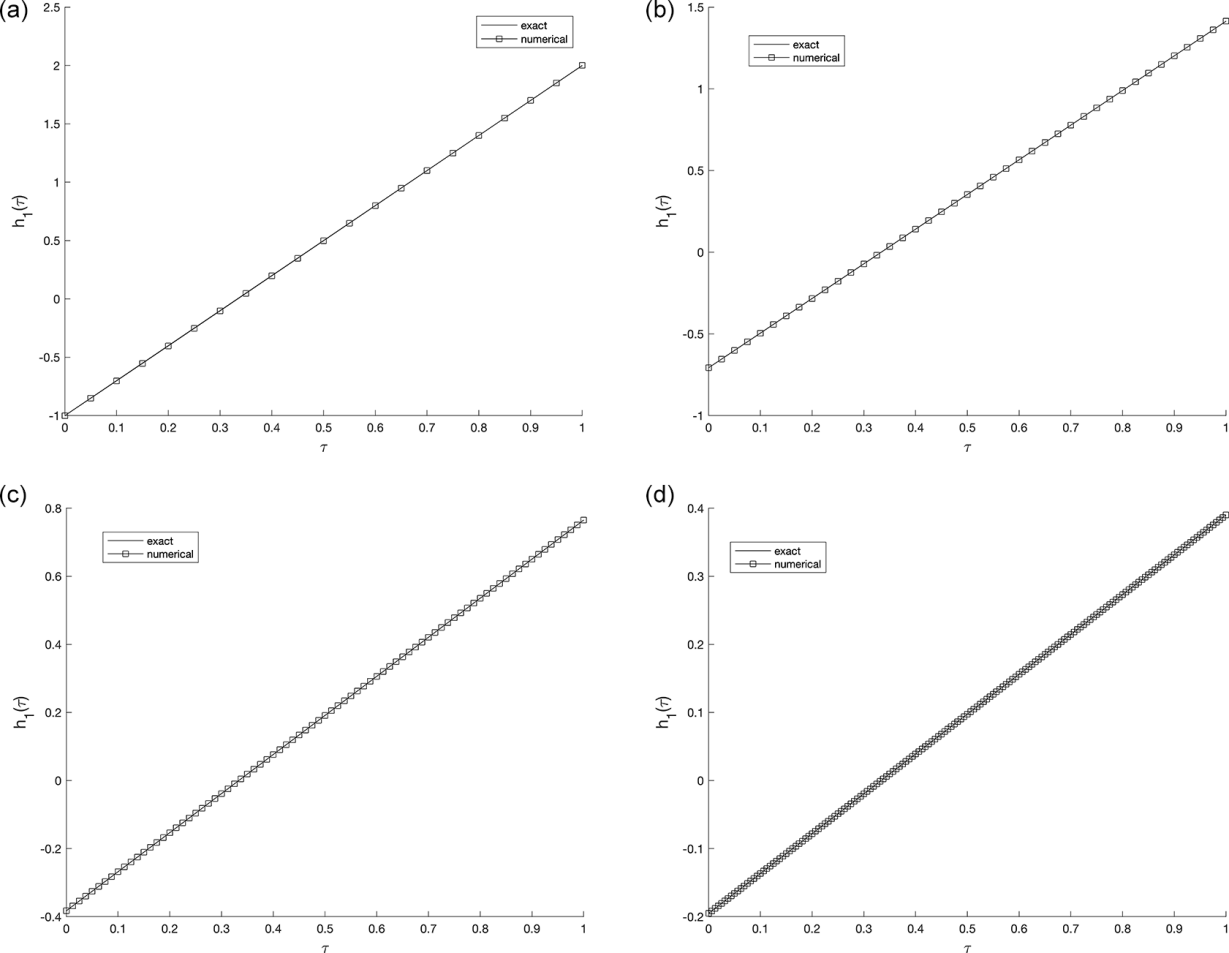
The graphs showing the exact and numerical values for, required output $w\left(v_{1},\tau \right)$ equation (5) , for example 1 (a2)−(d2), with the size of mesh N=M={20,40,80,160} , respectively.

**Figure 3.**
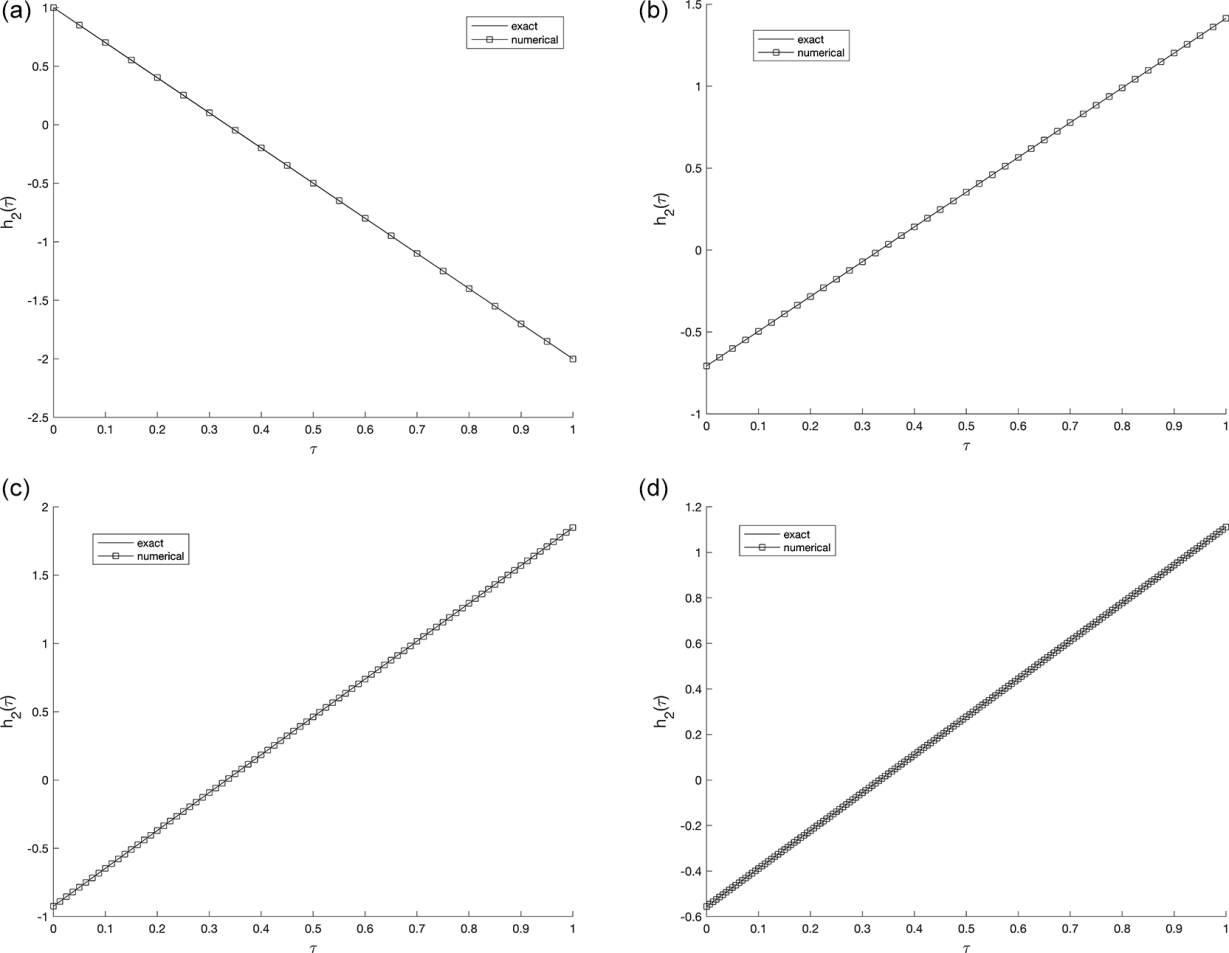
The graphs showing the exact and numerical values for, required output $w\left(v_{2},\tau \right)$ equation (5) , for example 1 (a3)−(d3), with the size of mesh N=M={20,40,80,160} , respectively.

The obtained results clearly indicate that the mesh size has a noticeable influence on the numerical accuracy. As the spatial and temporal mesh are refined (i.e., as M and N increase), the numerical solution becomes smoother and shows a closer agreement with the analytical one. This behavior confirms the expected second-order convergence of the C-N finite difference scheme in both time and space. Beyond a certain refinement level (e.g., M=N≥80 ), further mesh subdivision produces only negligible changes, implying that the proposed scheme is numerically stable and nearly mesh-independent. Whilst, Figure 2 and Figure 3, show the numerical outcomes for the required information $w\left(v_{i},\tau \right),\left(i=1,2\right),$ evaluated at various mesh sizes such that (s.t.), observed that an excellent agreement is obtained.

**3.2.2 Example 2:** Assume an illustration for the direct problem given by (1)−(4) with $D_{T}:\left\{\;0\;\leq \;v\;\leq \;1,0\;\leq \;\tau \;\leq \;1\;\right\},$ and the input data are,

$$f\left(v,\tau \right)=\frac{1}{2}e^{\tau }\left(\left(2v-1\right)\left(6+\left(5+\tau \right)\left(v-1\right)v\right)-\frac{2\mathrm{Sin}\left(\mathrm{\pi v}\right)}{1+\tau }\right),z\left(\tau \right)=\frac{-3e^{2\tau }}{32\sqrt{2}},c\left(\tau \right)=1,$$

and, the analytical solution is,

$$w\left(v,\tau \right)=e^{\tau }\left(1-v\right)\left(v-\frac{1}{2}\right)v,g\left(v,\tau \right)=e^{\tau }\mathrm{Sin}\left(\mathrm{\pi v}\right),a\left(\tau \right)=6+\tau ,\text{with},b\left(\tau \right)=\frac{1}{\left(1+\tau \right)},\left(v_{1},v_{2}\right)=\left(\frac{1}{4},\frac{3}{4}\right),\tau \;\in \;\left[0,T\right],\left(v,\tau \right)\;\in \;D_{T}\;$$

**Figure 4.**
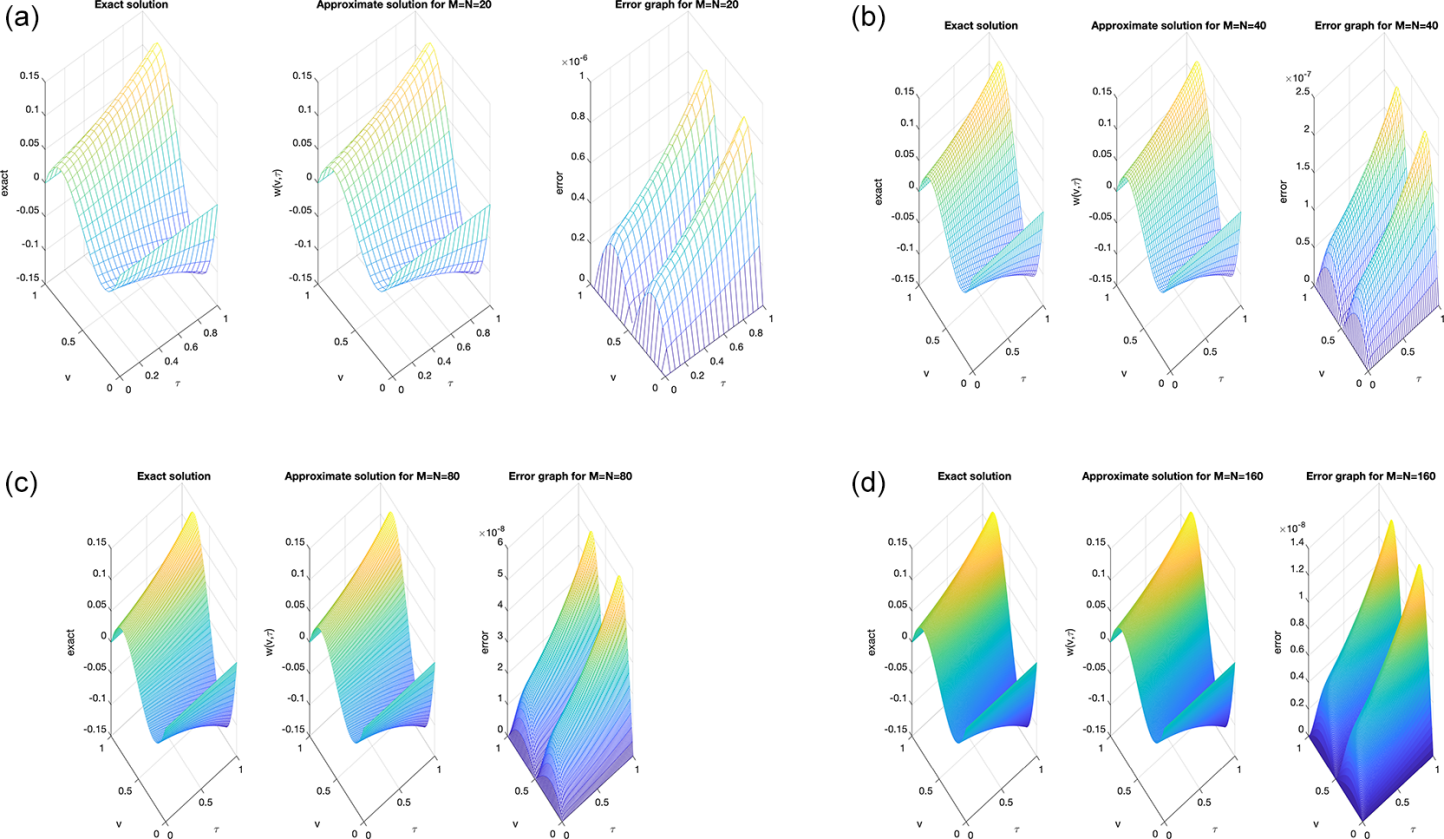
The graphs showing analytical and computational distribution with absolute error for the direct problem (1)−(4) , for example2 (a4)−(d4), represent the size of mesh N=M={20,40,80,160} , respectively.

The accuracy of the numerical solution is examined using several mesh grid size, M=N∈{20,40,80,160} . Figure 4 (a)–(d), clearly demonstrates an excellent agreement between the analytical and numerical solutions, confirming the reliability of the proposed approach. It can be noted that as the number mesh becomes finer, the numerical accuracy improves, indicating the expected convergence and stability of the C-N finite difference scheme. Furthermore, the absolute error plots at different mesh sizes reveal that the maximum error magnitude remains below $\left(10^{-8}\right)$ , as illustrated in Figure 4 (a)–(d).

**Figure 5.**
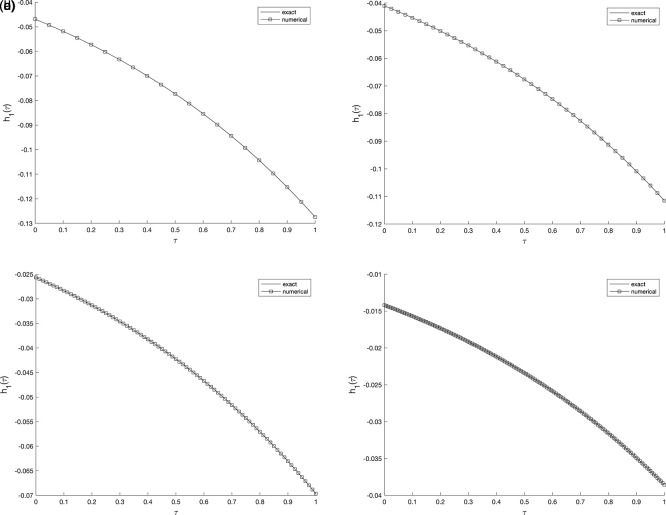
The graphs showing the exact and numerical values for, required output $w\left(v_{1},\tau \right)$ equation (5) , for example 2 (a5)−(d5), with the size of mesh N=M={20,40,80,160} , respectively.

**Figure 6.**
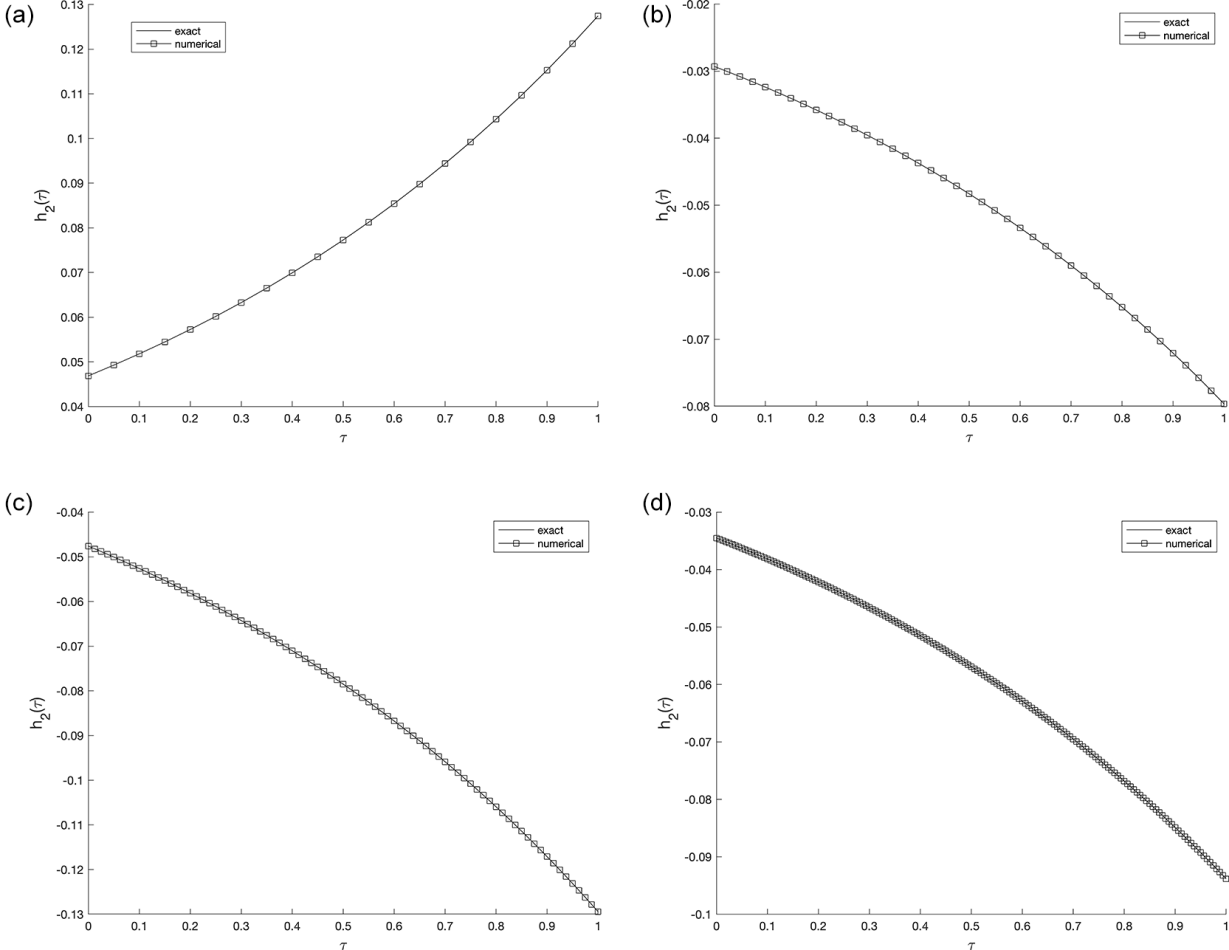
The graphs showing the exact and numerical values for, required output $w\left(v_{2},\tau \right)$ equation (5) , for example 2 (a6)−(d6), with the size of mesh N=M={20,40,80,160} , respectively.

Similarly, in Example 2, the refinement of the spatial and temporal mesh has a direct impact on the numerical accuracy. As the grid is successively refined, the computed solution aligns more closely with the analytical one, and the absolute error distribution becomes smoother across the domain. This confirms that the proposed C-N finite difference formulation maintains its second-order convergence and numerical stability. It can therefore be concluded that the results are consistent and nearly insensitive to further mesh refinement beyond the tested resolutions, indicating satisfactory mesh-independence of the method. Whilst, Figure 5 and Figure 6, show the numerical outcomes for the required information $w\left(v_{i},\tau \right),\left(i=1,2\right),$ evaluated at various mesh sizes s.t. observed that an excellent agreement is obtained.

## 4. Inverse problem solution for Equations (1)–(5)

For the nonlinear IP (1)−(5), we seek precise and stable identification of w(v,τ),a(τ) and b(τ) , that mean potential term a(τ) , and b(τ) is unknown. The one-dimensional second-order parabolic equation together with w(v,τ) satisfies the problem given by Equation (1)–(5), the problem is reformulated as a nonlinear least-squares minimization task. The resulting minimization problem is solved numerically using the *lsqnonlin* routine available in MATLAB’s optimization Toolbox, which implements a trust-region reflective algorithm suitable for nonlinear least squares problems with bound constraints. Due to the ill-posedness of such problems, especially in the presence of noise data, Tikhonov Regularization (TR) is employed to stabilize the solution. The regularization parameter β≥0 , is selected carefully to balance the trade-off between measured and the numerically computed solution. For this purpose, MATLAB routine *lsqnonlin* is employed, to minimize the Tikhonov Regularized objective functional, the procedure begins with a suitably selected initial guess. The TR functional is derived based on condition (5) , and the functional error is incorporated as follows:

$$F\left(a\right)=\sum_{i=1}^{2}{{\left\|\;w\left(v_{i},\tau \right)-z_{i}\left(\tau \right)\right\|}^{2}}_{L^{2}\left[0,\tau \right]}+\beta \left({{\left\|\;a\left(\tau \right)\right\|}^{2}}_{L^{2}\left[0,\tau \right]}+{{\left\|\;b\left(\tau \right)\right\|}^{2}}_{L^{2}\left[0,\tau \right]}\right),$$ (37)

where β>0, is a regularization parameter.

The unregularized case, i.e., β=0, produces the regular a nonlinear least-squares functional, which is inherently unstable when dealing with noisy data. The MATLAB routine *lsqnonlins* is used to minimize F under some physical constraint. The following parameters had been used for the subroutine:

• (Maxlter) maximum number of iterations $=10^{6}х\left(N\right)$ .
• Solution and Objective function tolerance $=10^{-10}$

• The lower and upper bounds on the component of the vector $\underline{a}$ are $-10^{-2}$ and $10^{2}$ , respectively.

The IP (1)−(5) are resolved via both precise and noisy measurement (5) . By including a random error, the noisy data is numerically simulated:

$$z^{\varepsilon }\left(\tau_{j}\right)=z\left(\tau_{j}\right)+\varepsilon_{j}\;j=\bar{0,N.}$$ (38)

where ε , is random Gaussian normal distribution vectors with mean zero and standard deviations σ , given by

$$\sigma =\rho \;х\;\max_{\tau \epsilon \left[0,T\right]}\left|z\left(\tau \right)\right|$$ (39)

where, ρ is the percentage of noise. We use the MATLAB function “ *normrnd*”, as:

$$\underline{\varepsilon }=\text{normrnd}\left(0,\sigma ,N\right),$$ (40)

to generate the random variables $\underline{\varepsilon }=\left(\varepsilon_{j}\right)$ and, $j=\bar{0,N.}$

## 5. Results and discussion

To evaluate the precision and stability of the numerical methods, a set of numerical experiments is conducted. These experiments are designed to evaluate the accuracy of the computed results by simulating realistic measurement conditions, where noise is introduced into the input data. To quantitatively evaluate the discrepancy between the exact and the numerically computed solutions, the root mean squares errors (RMSE) are utilized by the following expression

$$\text{RMSE}\left(a\right)=\sqrt{\frac{1}{N}\sum_{i=1}^{N}{\left(a_{i}^{\text{exact}}-a_{i}^{\text{numerical}}\right)}^{2}},$$ (41)

we take, T=1 for simplicity.

### 5.1 Example 3: Illustrates the solution of the IP together with the corresponding direct problem in order to demonstrate the effectiveness of the proposed regularization approach

Consider the IP (1)−(5) with the following input data,

$$f\left(v,\tau \right)=\left(-2+4\pi^{2}\left(3+\tau \right)-\tau \left(7+2\tau \right)\right)\mathrm{Sin}\left(2\mathrm{\pi v}\right)-\tau^{2}\left(7+2\tau \right)\mathrm{Sin}\left(4\mathrm{\pi v}\right),\tau \;\in \;\left[0,T\right],$$

$$z\left(\tau \right)=\frac{-1}{4}\sqrt{15+\sqrt{5\left(33-8\sqrt{2}\right)}}\;\left(3+\tau \right)\left(7+2\tau \right),c\left(\tau \right)=1,\tau \;\in \;\left[0,T\right],$$

where γ=2 , and, the analytical solution for this IP is provided as follows,

$$w\left(v,\tau \right)=\left(3+\tau \right)\mathrm{Sin}\left(2\mathrm{\pi v}\right),g\left(v,\tau \right)=\left(7+2\tau \right)\mathrm{Sin}\left(4\mathrm{\pi v}\right),a\left(\tau \right)=2\tau +1,\;\text{with},\;b\left(\tau \right)=\tau^{2},\left(v_{1},v_{2}\right)=\left(\frac{1}{8},\frac{2}{5}\right),\;\tau \;\in \;\left[0,T\right],\left(v,\tau \right)\;\in \;D_{T}$$

**Figure 7.**
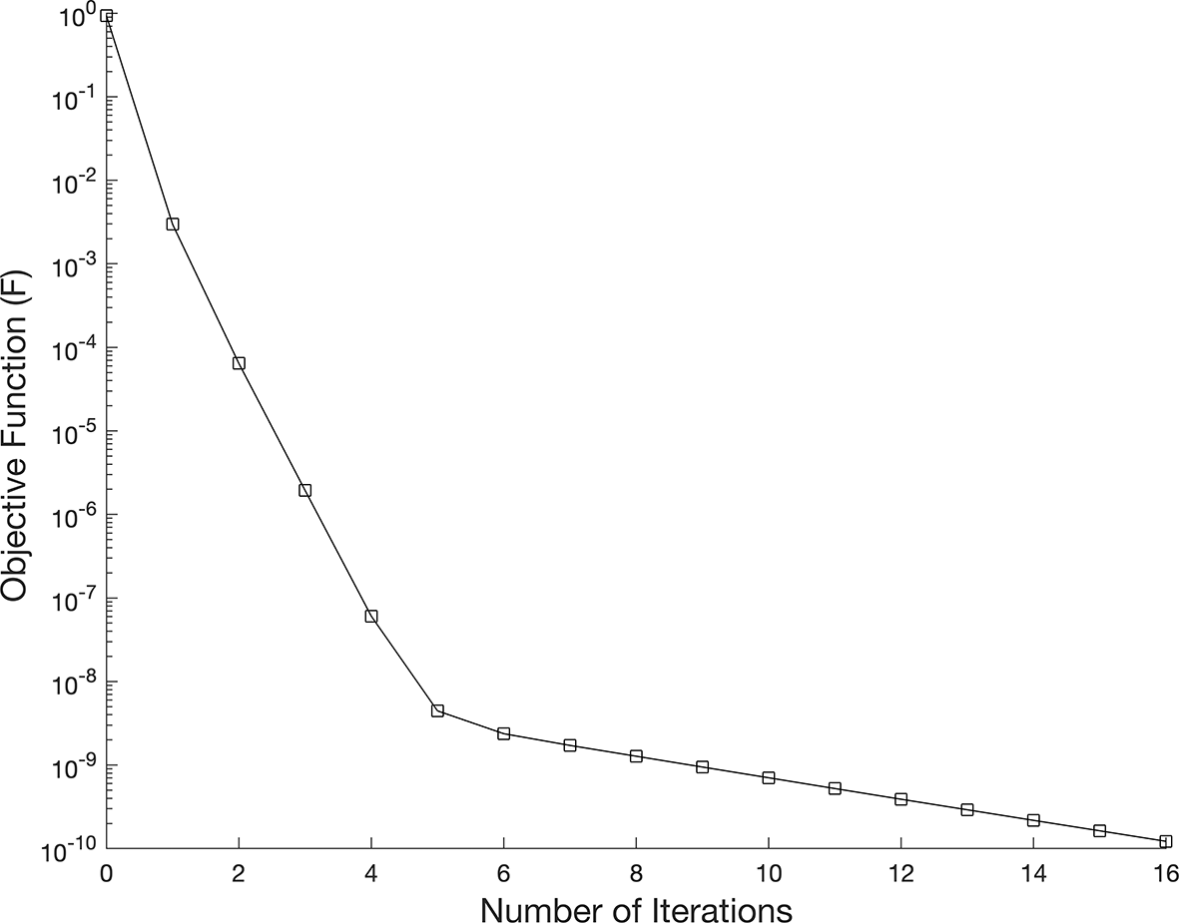
The graph showing the objective function for the IP (1)−(5) , in example 3 when the size of mesh N=M=80 .

The time-dependent coefficient a(τ) , and b(τ) , are then reconstructed (in case, M=N=80 , is taken), and consider the case of noise-free in the measurements, see Table 1, this mean p=0, in (37) and, no regularization applied, Figure 7 shows the objective function (37) without regularization i.e. β=0, while, in Figure 9, noise without regularization.

**Table 1.** Numerical reconstruction results for the IP showing the mesh size (M=N), root mean square error (RMSE) of the reconstructed coefficients and information for no regularization β=0 and noise p=0 .

M=N	*80*
**RMSE** a( τ )	0.0241
**RMSE** b( τ )	2.1592 $\times 10^{-4}$
**Computational time (second)**	3594 Sec
**Number of Iterations**	16
**Objective Function value**	1.2221× $10^{-10}$

**Figure 8.**
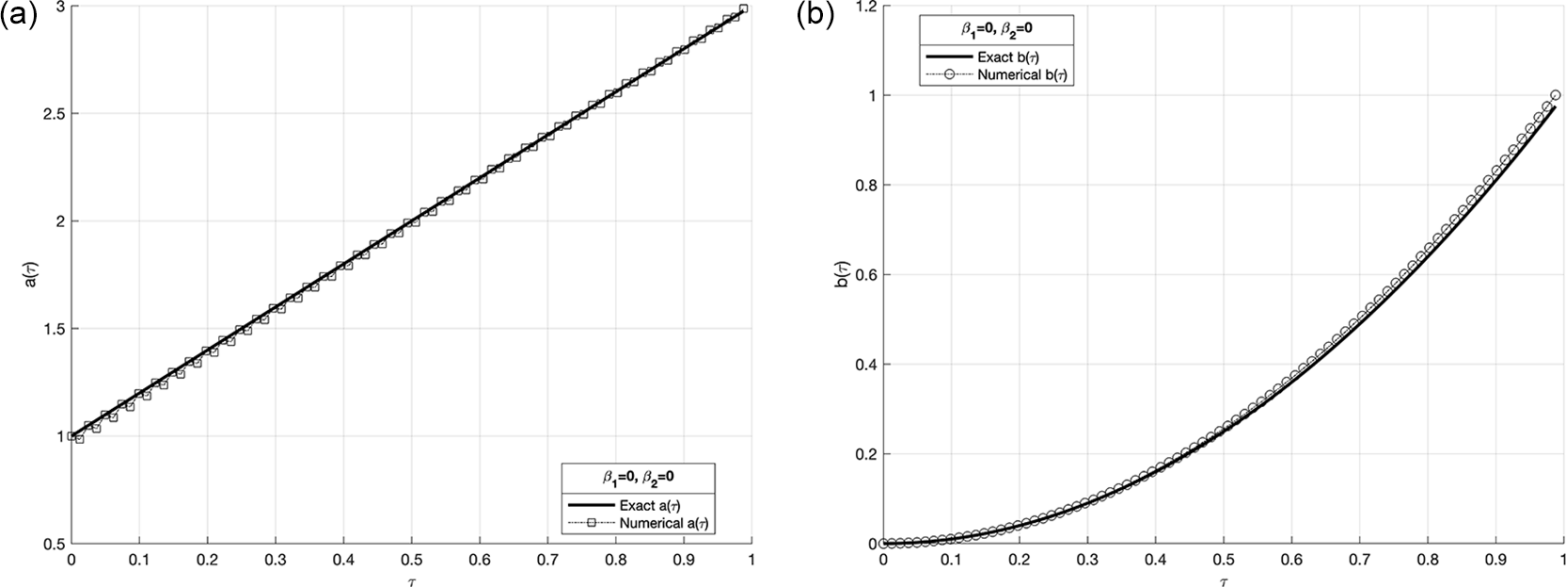
The graphs (a8) and (b8) showing the reconstructed coefficient a(τ) and b(τ) in comparison with exact value for the IP (1)−(5) , in example 3 when the size of mesh N=M=80 .

**Figure 9.**
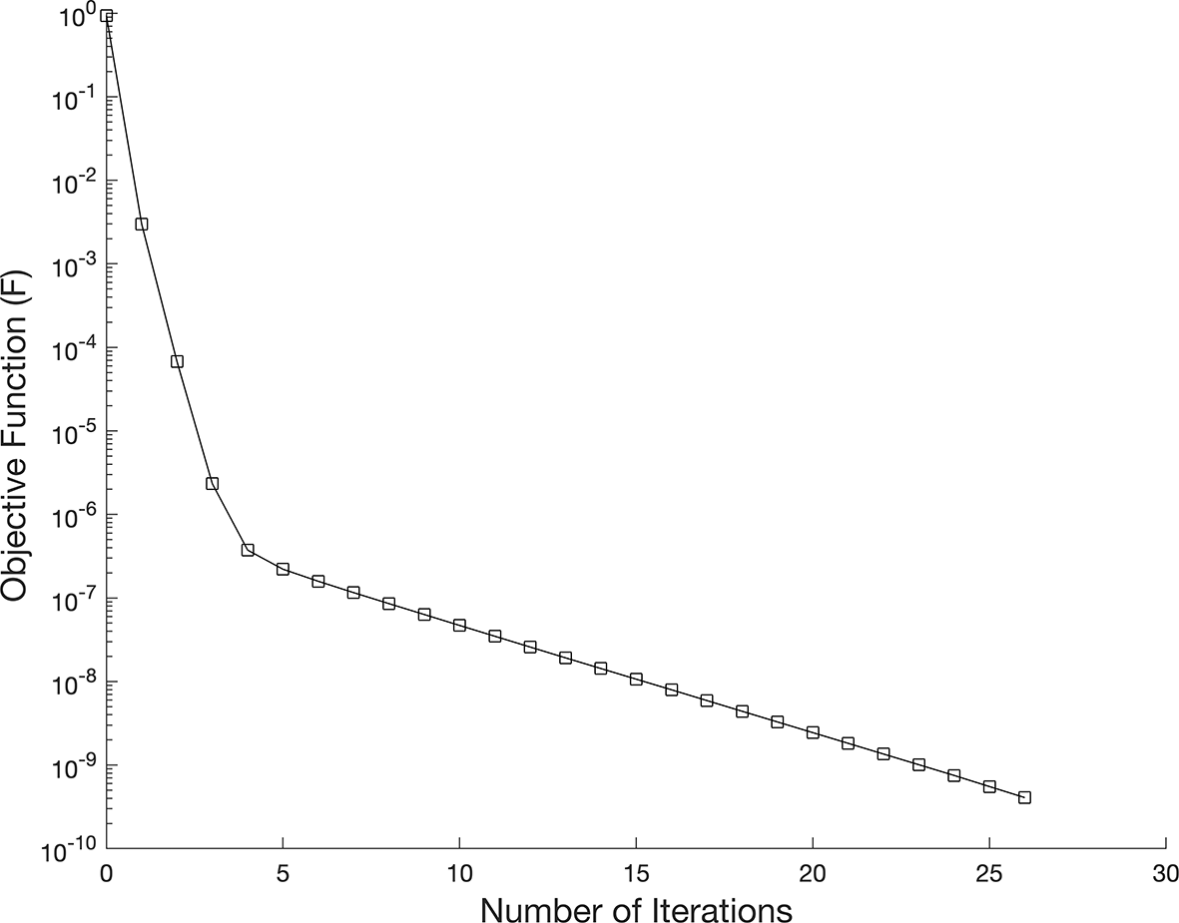
The graph showing the objective function when p=0.01%, no regularization for the IP (1)−(5) , in example 3, when the size of mesh N=M=80 .

**Figure 10.**
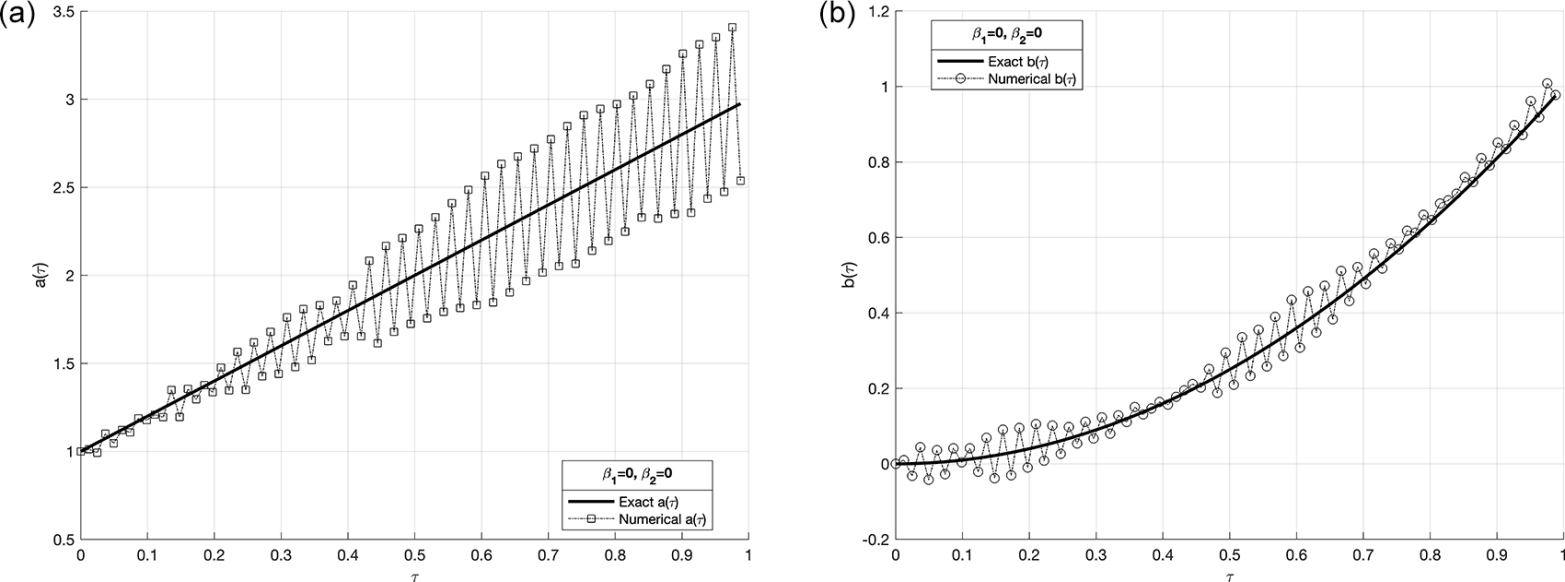
The graphs (a10) and (b10) showing the exact and (unstable) numerical solution for a(τ) and b(τ) when p=0.01%, no regularization for the IP (1)−(5) , in example 3, when the size of mesh N=M=80 .

**Figure 11.**
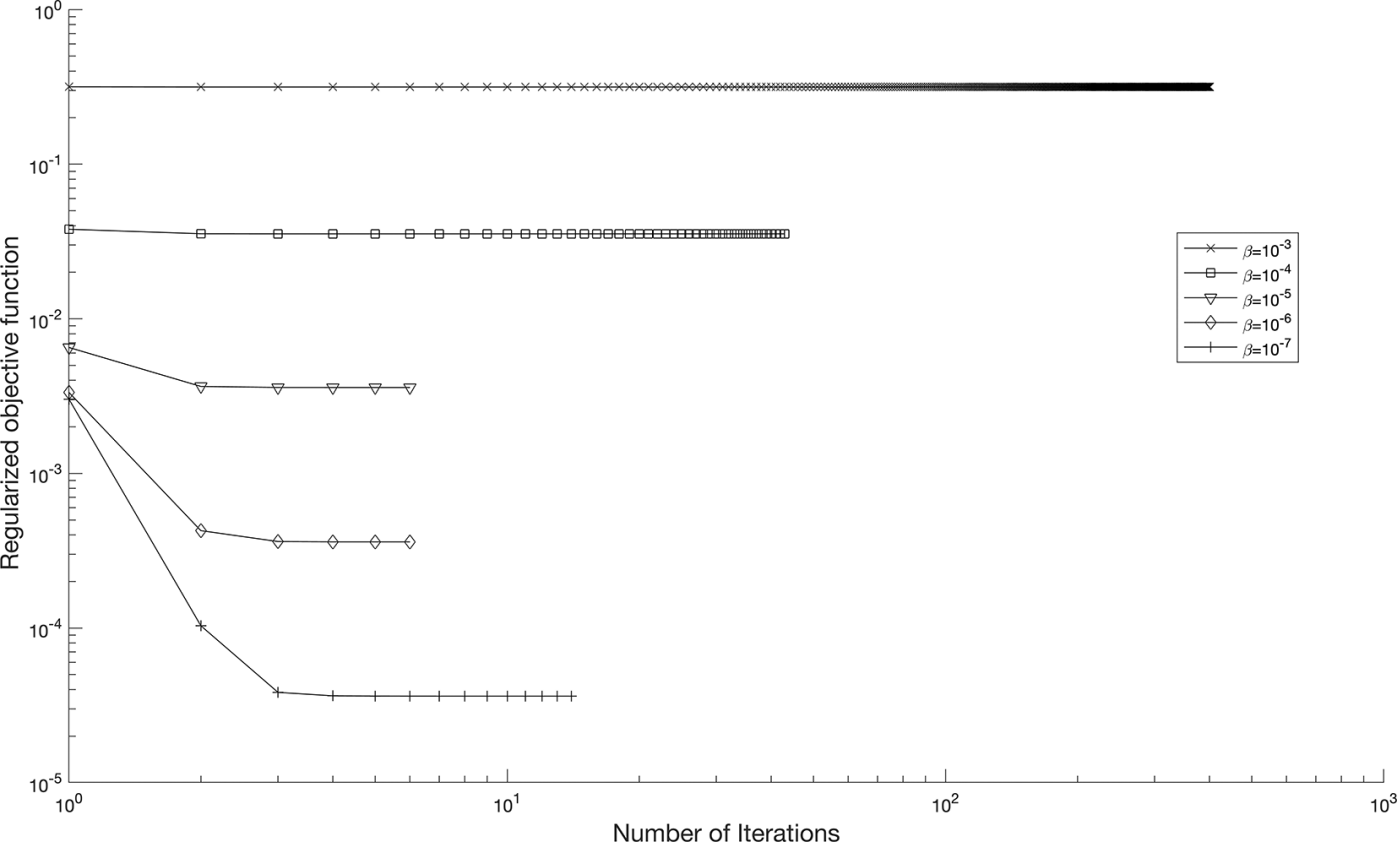
The graph showing the objective function when p=0.01% noise with regularization, $\beta \;\in \;\left\{10^{-3},\ldots ,10^{-7}\right\}$ , for the IP (1)−(5) , in example 3, when the size of mesh N=M=80 .

**Figure 12.**
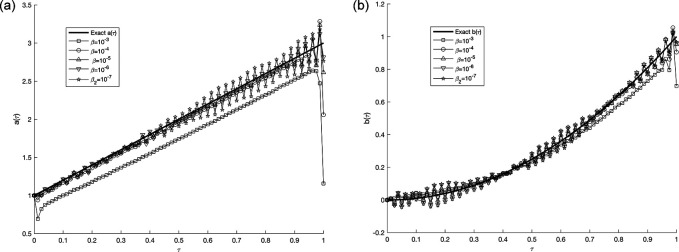
The graphs (a12) and (b12) showing the exact solution and regularized numerical reconstructions of a(τ) and b(τ) when p=0.01%, and several curves corresponding to different values of the regularization parameter $\beta \in \left\{10^{-3},\ldots ,10^{-7}\right\}$ are presented to illustrate the influence of the regularization parameter on the stability of the reconstructed solution, for the IP (1)−(5) , in example 3, when the size of mesh N=M=80 . All numerical computations were performed using MATLAB R2022a on a personal computer equipped with Intel Xeon E-2186M processor and 32 GB RAM, running windows 11 Pro for Workstations (64-bit). The computational complexity of the proposed algorithm mainly arises from solving the discretized parabolic equation at each iteration together with the nonlinear least-squares optimization procedure. The reported computational time corresponds to a single reconstruction run.

We investigate the time-dependent coefficient a(τ) and b(τ) under both exact and perturbed measurements, as demonstrated in Figures 7 − 12. The associated results are unstable, as illustrated in Figure 9- Figure 10. This reveals that the problem is not properly posed, a noticeable instability appears in the results, which is anticipated due to the ill-posed nature of the investigated IP. The unregularized solutions exhibit noticeable instability even in the noise-free case, demonstrating the sensitivity of the problem to small perturbations in the input data. The TR approach is used by incorporating the penalty term $\beta \left({{\left\|\;a\left(\tau \right)\right\|}^{2}}_{L^{2}\left[0,\tau \right]}+{{\left\|\;b\left(\tau \right)\right\|}^{2}}_{L^{2}\left[0,\tau \right]}\right)$ into the classical least-squares formulation, as presented in Equation (37). In Figure 11, p=0.01% noise strategy is employed to enhance the solution’s robustness with regularization parameters, $\beta \;\in \;\left\{10^{-3},\ldots ,10^{-10}\right\}$ , yielding numerically stable regularized reconstructions of a(τ) and b(τ) , as shown in Table 2, where the results for the case with noise level p=0% are provided in Table 1. In the following figure, Figure 12 illustrates the influence of different values of the regularization parameter *β* on the reconstructed coefficients *a*( *τ*) and *b*( *τ*) in the presence of noisy data. The results show that the regularization method stabilizes the solution, with smaller values of β proving effective when the data is not contaminated.

**Table 2.** Numerical results for different values of the regularization parameters with noise p=0.01%,(N=80 ) and grid size.

β	$10^{-3}$	$10^{-4}$	$10^{-5}$	$10^{-6}$	$10^{-7}$	$10^{-8}$	$10^{-9}$	$10^{-10}$
**RMSE a(** τ **)**	0.3302	0.1220	0.0714	0.0617	0.1189	0.2408	0.2835	0.2865
**RMSE b(** τ **)**	0.0552	0.0225	0.0259	0.0344	0.0365	0.0373	0.0377	0.0377
**Computational time (second)**	84320 Sec	9211 Sec	1453 Sec	1451 Sec	3139 Sec	6277 Sec	5814 Sec	5506 Sec
**Number of Iterations**	401	43	6	6	14	22	22	22

Next, we will study the proportion of noise contamination with a ratio of p=0.01% noise, in (38) via (39) , the associated results are unstable, as illustrated. This reveals that the problem is not properly, posed and small error in the data input cause large errors in the output solution, thus, the problem needs to be regularized. As a consequence, for the situation of p=0.01% noise, the best selection for the regularization parameters is $10^{-6}$ for a(τ) and $10^{-4}$ for b(τ) as in Table 2, which results in the lowest RMSE values.

## 6. Conclusions

In this study, the identification of the unknown time-dependent coefficient a(τ) and b(τ) in a one-dimensional parabolic PDE are investigated. The problem is formulated from a numerically evaluated nonlocal integral subjected to initial and boundary conditions. The direct problem is solved using the Crank-Nicolson (C-N) scheme, while the IP is transformed into a nonlinear least squares optimization problem using the *lsqnonlin* routine in MATLAB. An example test cases are employed in numerical experiments to evaluate the accuracy and stability of the proposed method. According to the results, using TR significantly enhances the solution’s stability. When the root-mean square error (RMSE) numbers are their lowest, the most effective approach is identified. Furthermore, numerical results have been provided to illustrate the accuracy and stability of the numerical method. It found that applying TR method stabilizes the solution. Cases of numerical results exist with noise (with and without) regularization. In the first case, consider when p=0.01% noise and without regularization as in Figure 9- Figure 10 in example 3, the results are unstable and highly oscillated, on the contrary. Other case, when p=0.01% noise introduced with $\beta \;\in \;\left\{10^{-3},\ldots ,10^{-10}\right\},$ regularization, obtain are stable and accurate results for the reconstructed coefficient a(τ) and b(τ) , which are (stable) numerical solutions. As for the remaining cases, we observe that the results are stable and steady when regularization values are $10^{-6}$ for a(τ) and, $10^{-4}$ for b(τ) in example 3, as shown in Table 2.

## Data Availability

Data sharing not applicable to this article as no datasets were generated or analyzed during the current study.
