# Supplementary material for: Simultaneous Numerical Determination of Two Time-dependent Coefficients in Second Order Parabolic Equation With Nonlocal Initial and Boundary Conditions
Source: F1000Res. 2026 Apr 3;15:228. Originally published 2026 Feb 10. [Version 2] doi: 10.12688/f1000research.173252.2 (PMC12966797; doi:10.12688/f1000research.173252.2)
Supplement: Peer review history

# Reviewer report: Reviewer response for version 2

**DOI:** 10.5256/f1000research.197752.r472944

**Published:** 2026-04-08

**Authors:** Areena Hazanee

**Affiliations:** 1 Prince of Songkla University, Hat Yai, Thailand

The manuscript has been suitably revised in accordance with the suggestions.

Is the work clearly and accurately presented and does it cite the current literature?

Yes

If applicable, is the statistical analysis and its interpretation appropriate?

Not applicable

Are all the source data underlying the results available to ensure full reproducibility?

Yes

Is the study design appropriate and is the work technically sound?

Yes

Are the conclusions drawn adequately supported by the results?

Yes

Are sufficient details of methods and analysis provided to allow replication by others?

Yes

Reviewer Expertise:

Inverse problem

I confirm that I have read this submission and believe that I have an appropriate level of expertise to confirm that it is of an acceptable scientific standard.

# Reviewer report: Reviewer response for version 1

**DOI:** 10.5256/f1000research.191048.r458509

**Published:** 2026-03-09

**Authors:** Areena Hazanee

**Affiliations:** 1 Prince of Songkla University, Hat Yai, Thailand

The manuscript presents a numerical method for solving an inverse problem to determine two coefficients. The Crank-Nicolson finite difference method, combined with Tikhonov regularization, is employed. The MATLAB toolbox ‘lsqnonlin’ is used to solve the nonlinear regularized least-squares optimization problem. Two numerical examples are provided for the direct problem, and one numerical example is presented for the inverse problem.

 This paper is interesting as it addresses the simultaneous estimation of two unknown time-dependent coefficients in an inverse problem. The manuscript includes numerical examples for both the direct and inverse problems, which help demonstrate the effectiveness of the proposed method. Therefore, the manuscript is recommended for indexing.

 Please find the attachment for the recommendation for revision.

**Review**

1. Section 2, Page 4 : The theorem on the existence and uniqueness of the inverse problem is presented with reference [28]. However, reference [28] is missing from the reference list.
2. Section 2, Page 6 : This section discusses the existence and uniqueness of the inverse problem
  (1)–(5). However, the theorem statement refers only to problems (1)–(3), mentioned at the end of the theorem statement. Please verify this with the cited reference.
3. Section 3, Page 6 : In this inverse problem, the unknown functions are a(τ) and b(τ), while c(τ) is assumed to be known in both the direct and inverse problems. However, in the first line of
  Section 3, c(τ) is described as an unknown function that is taken to be known for the direct problem. This statement may confuse the reader and should be clarified as ‘i.e., when unknown a(τ) and b(τ) are assumed to be given’.
4. Section 3, Page 7 : Please recheck Equation (34) together with the definitions of L, L1, V and W.
5. Section 3.2 : Although the main focus of the paper is the inverse problem, two numerical examples are presented for the direct problem, while only one example is given for the inverse problem. It would be beneficial to include at least one example addressing both the direct and inverse problems in order to demonstrate how the regularization method retrieves the unknown coefficients.
6. Section 3.2, Page 15, Figure 8 : The figure does not appear on the same page where it is referenced. Please indicate the example number for Figure 8.
7. Section 3.2, Page 15, Figure 9 : The figure was mentioned in Example 3, not Example 2. Please correct it.
8. Section 3.2, Page 16, Figure 12 : There are too many curves corresponding to different parameter values, which makes the figure difficult to read. It is recommended to present only the most representative parameter case and compare it with the no-noise case in order to better illustrate how the regularization method retrieves the unstable solution.
9. Section 3.2, Page 16, Table 2 : It would be helpful if Table 2 also included results without noise for comparison, in order to better demonstrate the effect of the regularization method.

Is the work clearly and accurately presented and does it cite the current literature?

Yes

If applicable, is the statistical analysis and its interpretation appropriate?

Not applicable

Are all the source data underlying the results available to ensure full reproducibility?

Yes

Is the study design appropriate and is the work technically sound?

Yes

Are the conclusions drawn adequately supported by the results?

Yes

Are sufficient details of methods and analysis provided to allow replication by others?

Yes

Reviewer Expertise:

Inverse problem

I confirm that I have read this submission and believe that I have an appropriate level of expertise to confirm that it is of an acceptable scientific standard.

# Response

**Published:** 2026-03-14

**Authors:** Mohammed Sabah Hussein

Dear Dr. Areena Hazanee 

**We sincerely thank the reviewer for their careful reading and valuable comments.**

**All of the suggested corrections have been incorporated into the revised manuscript. Below are the detailed responses to each of the reviewers' comments.**

1. **The citation [28] has been corrected to [27] in the revised manuscript to ensure consistency with the references list.**
2. **The statement of the theorem has been updated to refer to problem (1)-(5)**
3. **The first sentence of Section 3 has been revised to clarify that the coefficients** **a(τ)** **, c(τ) and b(τ)** **are assumed to be given.**
4. **The definitions and equation have been rechecked and revised for accuracy. The corrected expressions are now consistent and correctly defined before equation (34).**
5. **The wording for Example 3 has been revised to better highlight that it demonstrates both the direct problem and the corresponding inverse problem. The revised sentence is: ''Example 3 illustrates the solution of the IP together with the corresponding direct problem in order to demonstrate the effectiveness of the proposed regularization approach.''**
6. **The caption of Figure 8 has been revised as follows: ''Figure 8. The graphs (a8) and (b8) showing the reconstructed coefficients** **a(τ)** **and b(τ)** **in comparison with the exact value for the IP (1)-(5) in Example 3 when the mesh size N=M=80.**
7. **Thank you for pointing this out. The reference to Figure 9 has been has been corrected to Example 3, as it was indeed intended for this example. **
8. **The caption for Figure 12 has been updated to clarify the influence of different values of the regularization parameter** **β on the** **reconstructed coefficients** **a(τ) and b(τ)** **in the presence of noisy data. The revised caption is as follows: ''Figure 12. The graphs (a12) and (b12) showing the exact solution and regularized numerical reconstructions of** **a(τ) and b(τ) when p=0.01% and several curves corresponding to different values of the regularization parameter** **β** **∈** **{10−3,…,10−7}** **, are presented to illustrate the influence of the regularization parameter on the stability of the reconstructed solution for the IP (1)-(5) in Example 3 when the mesh size N=M=80.**
9. **The results for the case with noise level p=0% have been presented in Table 1 . Therefore, Table 2, focuses on the case with p=0.01% to illustrate the impact of the regularization technique on the solution.**

Best Regards

# Reviewer report: Reviewer response for version 1

**DOI:** 10.5256/f1000research.191048.r458506

**Published:** 2026-03-06

**Authors:** Raad Awad Hameed

**Affiliations:** 1 Tikrit University, Tikrit, Iraq

The paper investigates an interesting and highly relevant topic in the field of inverse problems, with practical applications in heat transfer and diffusion processes. The combination of the Crank-Nicolson method for the forward (Direct) problem and Tikhonov regularization for the inverse (indirect) problem is a mathematically sound approach. The numerical examples provided (Examples 1, 2 (for direct problem), and 3 (for inverse problem)) effectively demonstrate the proposed method's capability to reconstruct the unknown coefficients even when the input data is perturbed by noise.

 Below some points of the evaluation of the manuscript

  

- **In Abstract section:** In the Methods subsection, the phrase "efficiently solved used the MATLAB subroutine" contains a grammatical error and should be corrected to "efficiently solved *using* the MATLAB subroutine

- **In Introduction section:** There are several run-on sentences that impede clarity. For instance, "Compared with direct problems, IPs, are often more challenging to solve due to their ill-posedness, solutions may fail to exist, may not be unique, or may not change continuously with the input data". This should be split into multiple sentences.
- **Punctuation and Spacing:** The manuscript suffers from excessive and incorrect comma usage (e.g., "In this paper, the article is divided into five sections, section 2, includes a mathematical formulation..."). A thorough language copy-edit is needed.
** **
- **In Tables:** The text refers to "Table 1" and "Table 2" summarizing the numerical information, RMSE, and computational time. Ensure that the captions in the final manuscript clearly define the units for computational time (e.g., seconds) and that all abbreviations (like "Obj. Function") are introduced properly.
- before the equation 3   with periodic boundary conditions in the spatial variable *v* on the interval** ,

Overall, I recommend the acceptance of manuscript after addressing the minor changes.

 Sincerely,

  Dr. Raad A. Hameed

 Reviewer

Is the work clearly and accurately presented and does it cite the current literature?

Yes

If applicable, is the statistical analysis and its interpretation appropriate?

Yes

Are all the source data underlying the results available to ensure full reproducibility?

Yes

Is the study design appropriate and is the work technically sound?

Yes

Are the conclusions drawn adequately supported by the results?

Yes

Are sufficient details of methods and analysis provided to allow replication by others?

Yes

Reviewer Expertise:

partial differential equations

I confirm that I have read this submission and believe that I have an appropriate level of expertise to confirm that it is of an acceptable scientific standard.

# Response

**Published:** 2026-03-14

**Authors:** Mohammed Sabah Hussein

**Dear Prof. Dr. Raad A. Hameed**

**We sincerely thank the reviewers for their careful reading and valuable comments.**

**All of the suggested corrections have been incorporated into the revised manuscript. Below are the detailed responses to each of the reviewers' comments.**

1. **The sentence has been corrected in the revised manuscript to: ''efficiently solved using the MATLAB subroutine *lsqnonlin *from the MATLAB optimization Toolbox''.**
2. **The introduction section has been carefully revised and long run-on sentences have been split into shorter sentences to improve clarity and readability.**
3. **The manuscript has been carefully edited to correct punctuation and spacing issues. Unnecessary commas have been removed and several sentences have been rewritten to improve grammatical accuracy and readability.**
4. **The captions of Table 1 and Table2 have been revised to clearly indicate the unit of computational time (second). In addition, all abbreviations used in the Tables, including RMSE and objective function, have been properly defined in the revised manuscript.**

**The description preceding Equation (3) has been revised to clearly state the periodic boundary conditions in the spatial variable v over the specified interval.**

**Best Regards**

# Reviewer report: Reviewer response for version 1

**DOI:** 10.5256/f1000research.191048.r458507

**Published:** 2026-03-06

**Authors:** Ibrahim Tekin

**Affiliations:** 1 Alanya Alaaddin Keykubat University, Antalya, Turkey

The manuscript studies the simultaneous numerical identification of two time-dependent coefficients in a second-order parabolic equation subject to nonlocal initial and boundary conditions. Inverse coefficient problems for parabolic equations are well known to be ill-posed and mathematically challenging, and the simultaneous reconstruction of multiple time-dependent parameters represents a nontrivial extension of standard inverse formulations. Therefore, the topic is relevant and potentially valuable for the inverse problems and applied mathematics community.

 The manuscript presents a numerical reconstruction framework and provides computational experiments intended to demonstrate the effectiveness of the proposed approach. While the general structure of the paper is acceptable, several issues should be addressed in order to improve the scientific clarity, contextualization within the existing literature, and transparency of the computational results.

 The following points should be considered by the authors:

 Language and presentation:

 The manuscript would benefit from careful language editing. Several sentences are grammatically unclear or stylistically weak, which occasionally makes the mathematical arguments difficult to follow.

 Reference correction:

 On page 4, reference [28] appears to be incorrectly cited and should be corrected to [27].

 Literature context:

 The Introduction currently provides a limited overview of the recent literature on inverse coefficient problems for parabolic equations. The authors should expand the literature review to better position their contribution relative to existing numerical and analytical studies, particularly those addressing nonlocal boundary conditions and overdetermination data.

 Computational efficiency:

 In Table 1, the reported computational time is approximately 3594 seconds for only 16 iterations. For a one-dimensional parabolic inverse problem, this computational cost appears relatively high. The authors should therefore:

 1.Specify the hardware and software environment used for the computations.

 2.Provide a brief discussion of the computational complexity of the proposed algorithm.

 3.Clarify whether the reported time corresponds to a single reconstruction or multiple runs.

 Addressing these points would substantially improve the clarity and scientific positioning of the manuscript.

 Suggested Additional References (for the Introduction/Literature Review)

 Huzyk, et al., 2023 - Ref 1.

 Azizbayov, et al., 2025 - Ref 2.

 These studies may help broaden the discussion of related inverse coefficient problems and numerical approaches.

 The manuscript addresses a relevant inverse problem and proposes a numerical approach that may be of interest to researchers working on inverse parabolic problems. However, several clarifications and moderate revisions are required before the article can be considered fully scientifically sound. The requested revisions mainly concern improvements to the literature review, language clarity, and the discussion of computational aspects.

Is the work clearly and accurately presented and does it cite the current literature?

Partly

If applicable, is the statistical analysis and its interpretation appropriate?

I cannot comment. A qualified statistician is required.

Are all the source data underlying the results available to ensure full reproducibility?

No source data required

Is the study design appropriate and is the work technically sound?

Yes

Are the conclusions drawn adequately supported by the results?

Yes

Are sufficient details of methods and analysis provided to allow replication by others?

Yes

Reviewer Expertise:

Invese problems, Applied Mathematics

I confirm that I have read this submission and believe that I have an appropriate level of expertise to confirm that it is of an acceptable scientific standard, however I have significant reservations, as outlined above.

## References

[1] : Coefficient inverse problem for the strongly degenerate parabolic equation. *Carpathian Mathematical Publications* .2023;15(1) : 10.15330/cmp.15.1.52-65 52-65 10.15330/cmp.15.1.52-65

[2] : An Inverse Problem for a Parabolic Equation with Nonlocal Boundary Conditions and Two-point Overdetermination. *European Journal of Pure and Applied Mathematics* .2025;18(4) : 10.29020/nybg.ejpam.v18i4.6796 10.29020/nybg.ejpam.v18i4.6796

# Response

**Published:** 2026-03-14

**Authors:** Mohammed Sabah Hussein

**Dear Dr. Ibrahim Tekin**

**We sincerely thank the reviewer for their careful reading and valuable comments.**

**All of the suggested corrections have been incorporated into the revised manuscript. Below are the detailed responses to each of the reviewers' comments.**

1. **The manuscript has been carefully revised to improve language clarity and readability. Several sentences throughout the introduction and numerical methodology sections have been edited.**
2. **The incorrect citation on page 4 has been corrected from [28] to [27]**
3. **The introduction has been expanded to include additional recent studies on inverse coefficient problems for parabolic equations with nonlocal boundary conditions. In particular, the works of Huzyk et al. (2023) and Azizbayov and Safarova (2025) have been incorporated.**

**The hardware and software environment used for the computations has been added in the numerical results section. A brief discussion of the computational complexity has also been included, and it has been clarified that the reported time corresponds to a single reconstruction run.**

**Best Regards**

# Reviewer report: Reviewer response for version 1

**DOI:** 10.5256/f1000research.191048.r458503

**Published:** 2026-02-28

**Authors:** Alla Tareq Balasim

**Affiliations:** 1 mathematics, college of basic education, Baghdad, Iraq

I have reviewed the manuscript entitled "Simultaneous Numerical Determination of Two Time-dependent Coefficients in Second Order Parabolic Equation With Nonlocal Initial and Boundary Conditions."

 The paper addresses an important problem in inverse partial differential equations and proposes a stable numerical framework using the Crank-Nicolson finite difference method combined with Tikhonov regularization. 

 Strengths: -

 The study presents a clear scientific objective and demonstrates the effectiveness of inverse problems (IPs) in reconstructing unknown coefficients.

 The numerical methodology is appropriate and shows reliable convergence and stability. 

 Results confirm the robustness of the approach under both exact and noisy data.

 Minor Corrections Required:

 1. The introduction contains several long and complex sentences that should be revised for clarity and readability.

 2. References (2–5) are not consistent in formatting and should be unified according to the journal’s style. 3. There is no reference 28.

 4. The stability and convergence are not rigorously proven. The authors are encouraged to provide a theoretical proof or cite appropriate references supporting these results.

 5. Equation 37 should be placed directly below (s a regularization parameter. The discretization form of (29) is).

 6. Figures 3-6 should be before section 4.

 Recommendation: The manuscript is scientifically sound and of clear value. I recommend acceptance after minor corrections.

 Best regards,

Is the work clearly and accurately presented and does it cite the current literature?

Yes

If applicable, is the statistical analysis and its interpretation appropriate?

I cannot comment. A qualified statistician is required.

Are all the source data underlying the results available to ensure full reproducibility?

No source data required

Is the study design appropriate and is the work technically sound?

Yes

Are the conclusions drawn adequately supported by the results?

Yes

Are sufficient details of methods and analysis provided to allow replication by others?

Yes

Reviewer Expertise:

Numerical analysis, Finite difference method, Group iterative method, Dynamical system.

I confirm that I have read this submission and believe that I have an appropriate level of expertise to confirm that it is of an acceptable scientific standard.

# Response

**Published:** 2026-03-14

**Authors:** Mohammed Sabah Hussein

**Dear Dr. Alaa Tariq**

**We sincerely thank the reviewer for their careful reading and valuable comments.**

**All of the suggested corrections have been incorporated into the revised manuscript. Below are the detailed responses to each of the reviewers' comments.**

  

1. **The introduction section has been carefully revised to improve clarity and readability. Several long and complex sentences have been rewritten and simplified while preserving the scientific meaning.**
2. **Thank you for pointing this out. References (2-5) have been revised and reformatted to ensure consistency with the journal's citation style.**
3. **The incorrect citation [28] has been corrected to [27] in the revised manuscript to ensure consistency with the references list.**
4. **A stability and convergence discussion has been included in section 3.1. In addition, appropriate references supporting the unconditional stability and second-order convergence of the Crank-Nicolson finite difference scheme have been added.**
5. **Equation (37) has been moved and placed directly below the sentence '' as a regularization parameter. The discretization from of (29) is'' to improve the logical flow of the presentation.**
6. **Figures 3-6 have been reposition al in the revised manuscript and are now placed before section 4 in order to maintain the correct sequence between the numerical illustration and the corresponding discussion.**

Best Regards
